# Singular boundary behaviour and large solutions for fractional elliptic equations

**DOI:** 10.1112/jlms.12692

**Published:** 2022-12-08

**Authors:** Nicola Abatangelo, David Gómez‐Castro, Juan Luis Vázquez

**Affiliations:** ^1^ Dipartimento di Matematica Alma Mater Universitá di Bologna Bologna Italy; ^2^ Mathematical Institute University of Oxford Oxford United Kingdom; ^3^ Instituto de Matemática Interdisciplinar Universidad Complutense de Madrid Madrid Spain; ^4^ Departamento de Matemáticas Universidad Autónoma de Madrid Madrid Spain

## Abstract

We perform a unified analysis for the boundary behaviour of solutions to nonlocal fractional equations posed in bounded domains. Based on previous findings for some models of the fractional Laplacian operator, we show how it strongly differs from the boundary behaviour of solutions to elliptic problems modelled upon the Laplace–Poisson equation with zero boundary data.

In the classical case it is known that, at least in a suitable weak sense, solutions of the homogeneous Dirichlet problem with a forcing term tend to zero at the boundary. Limits of these solutions then produce solutions of some non‐homogeneous Dirichlet problem as the interior data concentrate suitably to the boundary.

Here, we show that, for equations driven by a wide class of nonlocal fractional operators, different blow‐up phenomena may occur at the boundary of the domain. We describe such explosive behaviours and obtain precise quantitative estimates depending on simple parameters of the nonlocal operators. Our unifying technique is based on a careful study of the inverse operator in terms of the corresponding Green function.

## 1 INTRODUCTION

In recent years, there have been many studies on boundary value problems driven by nonlocal operators L obtained as fractional powers of uniformly elliptic operators, such as the Laplacian. In this context, according to the ‘degree of nonlocality’ of the leading operator in the differential equation, additional values need to be prescribed either on the boundary of the underlying domain or on its whole complement. So, given a regular bounded domain $\Omega \subseteq R^{n}$, the simplest Dirichlet problems take the form of an equation 

(1.1) Lu=finΩ,

complemented by homogeneous values

(1.2) $$u=0\;\text{on}\;\partial \Omega ,\;\text{or}\;\text{in}\;R^{n}\setminus \bar{\Omega },$$

the last choice depending on the nonlocal operator L. Sometimes, (1.1) is written in some weak form which also encodes (1.2). In the standard elliptic theory, (1.2) can be replaced by u=g on ∂Ω, for g an $L^{p}$ function and therefore a.e. finite on ∂Ω. In this paper, we study solutions to equations of the form (1.1) that develop an explosive behaviour at the boundary, that is, solutions satisfying

$$\begin{array}{c} u\left(x\right)\to +\infty ,\;\text{as}\;x\to x_{0},\;\text{for}\;\text{almost}\;\text{all}\;x_{0}\in \partial \Omega . \end{array}$$

They are usually called *large solutions* and they account for a new phenomenon, not appearing in the classical elliptic theory. We will show that large solutions are tightly connected to the solutions of the homogeneous problem via a natural limiting process. Finally, they exhibit quite peculiar divergence rates that we will derive. All of this will be done for a specific class of nonlocal operators L that includes the usual examples and more.

The present research is motivated by two striking results involving singular behaviour near the boundary for the solutions of (1.1) in the case where L is the so‐called restricted fractional Laplacian (RFL; for which one has to prescribe data on $R^{n}\setminus \bar{\Omega }$).

One of these striking results is the existence of nontrivial solutions of (1.1) such that f=0 in Ω which, moreover, are positive everywhere and blow up on the boundary. Explicit examples on the ball were constructed in [33] (see also [6, 8]). The existence of this kind of solutions was systematised independently in [30] (which also contains a thorough regularity theory, see also [29] for related results and [31] for a review) and in [1] for the RFL, and extended in [2] for the spectral fractional Laplacian (SFL; which requires prescribed data at the boundary). These correspond to positive harmonic functions in the theory of the standard Laplacian, although the classical theory does not admit any harmonic function with uniform blow‐up at the boundary.

The second striking result, described in [1] when L is the RFL and in [2] when L is the SFL, is that some admissible functions f produce solutions u blowing‐up at the boundary, although they are limits of solutions with ‘nice' f and zero boundary data. This is as well a new behaviour of the nonlocal problem, not present for the usual Laplacian.

For the case of the usual Laplacian, it is known that the wider classes of weak or very weak solutions obtained as limits of the variational solutions satisfy the boundary condition either in the sense of traces or in a more generalised sense, described in [36] as the average condition

(1.3) $$\eta^{-1}\int_{\left\{\mathrm{dist}(x,\partial \Omega )<\eta \right\}}|u|\to 0\;\;\text{as}\;\eta ↓0.$$

The aim of the present work is to show that these two blow‐up phenomena occur for a large class of nonlocal operators of elliptic type. We treat in a unified way the typical nonlocal elliptic equations, in particular the different fractional Laplacians on bounded domains. Our distinctive technique is based on the use of the Green kernel which gives a common roof to the several different cases. This approach extends previous work in [10, 28].

We consider a general family of operators indexed on two parameters: one describing the interior point singularity of the Green kernel, the other one the kernel's boundary behaviour. This requires serious technical work, that justifies the extension of the paper.

First, we want to study and classify the explosive (or *large*) solutions whose singularity is, in some sense, generated by the right‐hand side f. In particular, we compute explicitly the asymptotic boundary behaviour of u for the family of power‐like data $f≍\delta^{\beta }$ near the boundary, where δ(x):=dist(x,∂Ω). Here, we say that f≍g on a set if there exists C>0 such that $C^{-1}g⩽f⩽Cg$ on that set. Our main formula (1.5) gives the behaviour of u in terms of f and the kernel of L in simple algebraic terms. The formula covers the whole range of behaviours, explosive or not. We also translate estimate (1.3) to our context by introducing a suitable weight, taking care of the singular profiles (see Lemma 3.8). We provide some careful numerical computations, to show the formation of the boundary singularity due to the right‐hand side (see Figures 3 and 4).

Even if the solution operator for the Dirichlet problem acting on a class of good functions f produces solutions with Dirichlet boundary data, we show that the natural closure of that solution operator to its maximal domain of definition produces solutions which no longer satisfy the Dirichlet condition and could reach a range of boundary blow‐up that we describe. In the case of problems in which the boundary condition is set (for example, the SFL), this is counter‐intuitive. The occurrence of boundary blow‐up is a very important fact, that does not happen for the usual Laplacian.

Second, we remark how there is a different class of explosive solutions whose singularity is not generated by any right‐hand side. In fact, they can be chosen as ‘L‐harmonic in Ω’ in the sense Lw=0. This class relies on some hidden information in the form of singular behaviour that can be prescribed on the boundary. Moreover, this second class can be obtained as a limit of singular solutions of the previous class as the support of f concentrates at the boundary in a convenient way. This means they cannot be disregarded in any complete theory of the problem. See the detailed results in Section 4.

We conclude this introduction with an important remark. If a definition of solution of (1.1) is ‘too weak’, then the combination of the two classes seems to pose a problem to uniqueness, as it happens in the classical elliptic theory. This highlights the importance of a suitable definition of weak solution of (1.1) preserving uniqueness and including the classical solutions. We provide this definition in Section 2 under the name of *weak‐dual solution*, and show that the problem is then well‐posed. We also detect the optimal class of admissible data f. To take care of the second class, we construct a ‘singular boundary data’ problem. We give a well‐posed notion of solution for this second problem: uniqueness is the easy part.

### 1.1 Main topics and results

#### Existence and uniqueness results for (1.1)

To begin with, we need to produce a general existence theory for data f in good classes, that is, compactly supported and bounded. We want to treat a general class of operators such that the unique solution of (1.1)–(1.2) is given by the formula

(K0) $$G\left(f\right)\left(x\right)=\int_{\Omega }G\left(x,y\right)f\left(y\right)\;dy,\;\text{for}\;x\in \Omega ,$$

with kernels G:Ω×Ω→R such that, for any x,y∈Ω,

(K1) $$\begin{array}{cc} G(x,y) & =G\left(y,x\right),\;\partial \Omega \in C^{1,1},\;\text{and} \end{array}$$

(K2) $$\begin{array}{cc} G(x,y) & ≍\frac{1}{{\left|x-y\right|}^{n-2s}}{\left(\frac{\delta (x)\delta (y)}{{\left|x-y\right|}^{2}}\wedge 1\right)}^{\gamma }. \end{array}$$

The two exponents s and γ take values

(K3) $$\begin{array}{c} s,\gamma \in (0,1],\;\text{with}\;2s⩽n. \end{array}$$

Their relative values will play an important role in the results. Note that hypothesis (K1) means that G is self‐adjoint. This allows to cover a large class of self‐adjoint operators L, as we will describe in Subsection 1.2.

Throughout this note, we use the notation a∧b=min{a,b}, a∨b=max{a,b}.

The main structural assumption on G is (K2). This general assumption was introduced in [11] to cover some notable examples of this general class of operators given by the three most known fractional Laplacian operators:

(i) The RFL: in this case γ=s∈(0,1).
(ii) The SFL, for which γ=1 and s∈(0,1).
(iii) The regional or censored fractional Laplacian (CFL) which has γ=2s−1 and s∈(1/2,1).

These examples will be presented in some more detail in Subsection 1.2, so that we can adapt to them the general results. As we will explain, the estimate (K2) in each of these examples is recovered by *ad hoc* techniques in different papers. In Section 5, we will make a brief discussion on the general setting.

In Section 2, we prove existence, uniqueness, *a priori* estimates, and some regularity for problem (1.1). In Section 3, we prove that the optimal class of data f such that (K0) is well‐defined (meaning G(|f|)≢+∞) is

(1.4) $$f\in L^{1}\left(\Omega ,\delta^{\gamma }\right)=\left\{f\;\text{measurable}\;\text{in}\;\Omega :f\delta^{\gamma }\in L^{1}\left(\Omega \right)\right\}.$$

#### Boundary behaviour

As we mentioned, for the standard Laplacian −Δ, the zero boundary data are taken in some sense even when f is taken in the optimal class of data. The sense depends on how good is f, see the general results in [36]. A quite novel property of the RFL on bounded domains shows that this is not true for admissible f even if they are not so badly behaved. This is explained in [1] and we want to extend the analysis to our general class of operators and show the detailed relation between the operators, the boundary behaviour of f, and the singular boundary behaviour of the solution. The main information about the operators will be the values of γ and s.

**FIGURE 1:**
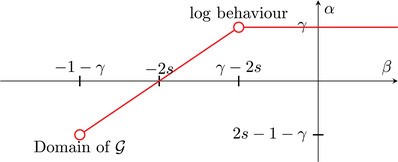
Relation of parameters α and β such that $G\left(\delta^{\beta }\right)≍\delta^{\alpha }$

In Theorem 3.4, we establish the explicit estimate

(1.5) $$G\left(\delta^{\beta }\right)≍\delta^{\gamma \wedge (\beta +2s)}\;\text{whenever}\;\gamma +\beta >-1\;\text{and}\;\beta \neq \gamma -2s$$

that needs a delicate computation using the properties of the kernel. This is depicted in Figure 1. Note that γ+β>−1 is the condition, so that $f=\delta^{\beta }$ belongs to the admissible class given by (1.4).

In many cases, the existence of eigenfunctions is known, and their boundary behaviour is well‐understood. Under (K0), (K2), and some extra assumptions on the operator L, the authors in [10] proved that the operator G admits an eigendecomposition and its first eigenfunction $\Phi_{1}$ satisfies

$$\Phi_{1}≍\delta^{\gamma }\;\text{in}\;\Omega .$$

The boundary behaviour is clear from the algebraic point of view, since γ is the only exponent fixed by G.

#### Solutions with singular behaviour

We observe that, according to formula (1.5), there are values of β for which the solution associated to datum $\delta^{\beta }$ is singular at the boundary: this happens whenever β∈(−1−γ,−2s) is allowed, and therefore when γ>2s−1. In particular, it comes out that if γ>2s−1, then there exist solutions of the Dirichlet problem not complying with the condition u=0 on the boundary. This was known for the RFL [1, Proposition 3] and the SFL [23, Proposition 7], whereas this phenomenon does not take place for the CFL (as we show below).

The behaviour $\delta^{\gamma \wedge (2s-\gamma -1)}$, corresponding to the limit case β=−1−γ, serves somehow as an upper bound for solutions. In Lemma 3.8, we will prove that

(a) if γ>s−1/2, then for any $f\in L^{1}\left(\Omega ,\delta^{\gamma }\right)$
$$\frac{1}{\eta }\int_{\left\{\delta <\eta \right\}}\frac{G(f)}{\delta^{2s-\gamma -1}}⟶0\;\text{as}\;\eta ↓0;$$
(b) if γ<s−1/2, then for any nonnegative $f\in L^{1}\left(\Omega ,\delta^{\gamma }\right)$ and η>0
$$\frac{1}{\eta }\int_{\left\{\delta <\eta \right\}}\frac{G(f)}{\delta^{\gamma }}≍1.$$

We also prove that, in the case γ=s−1/2, there is a logarithmic correction.

For the usual Laplacian, when s=γ=1, we have 0=2s−γ−1<γ: this reproduces (1.3). This same fact holds for the CFL, because γ=2s−1. If 2s−γ−1>0, then all solutions tend to 0 upon approaching the boundary.

**FIGURE 2:**
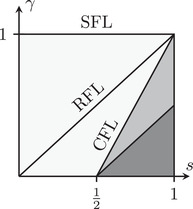
Different relations between γ and s

The two conditions γ>2s−1 and $\gamma >s-\frac{1}{2}$ allow us to split the parameter s,γ in three regions as in Figure 2.

#### Normal derivatives

A sharper study of the boundary behaviour of solution with data $f\in L_{c}^{\infty }\left(\Omega \right)$ consists of the analysis of the limit

$$D_{\gamma }u\left(z\right):=\lim_{\begin{array}{c} x\to z\\ x\in \Omega \end{array}}\frac{u(x)}{\delta {\left(x\right)}^{\gamma }},\;z\in \partial \Omega .$$

We will call this limit γ‐*normal derivative*. We devote Subsection 3.3 to the study of these normal derivatives (see Theorem 3.15).

#### Large solutions

In [8], the authors introduce a surprising singular solution of the homogeneous problem f=0 that shows very precise asymptotics at the boundary. It is the type known as *large solution* in other situations for nonlinear equations. For example, the function

(1.6) $$u\left(x\right):=\left\{\begin{array}{cc} {\left(1-{\left|x\right|}^{2}\right)}^{s-1} & \text{for}\;|x|<1\\ 0 & \text{for}\;|x|⩾1 \end{array}\right.$$

is known to satisfy ${\left(-\Delta \right)}_{\mathrm{RFL}}^{s}u\left(x\right)=0$ for |x|<1, see [6, Example 1] and [33]. In [1], there is a complete description of the singular boundary value problem for the RFL, while in [2] there is the analogue for the SFL. Note that, in the limit s→1, the u in (1.6) becomes the characteristic function of the ball.

We prove that this theory may be obtained as a limit of interior problems. We construct one such particular large solution $u^{★}$ which is L‐harmonic on the interior ($Lu^{★}=0$ in Ω). In Section 4, we show that there exists a sequence of admissible functions ${\left(f_{j}\right)}_{j\in N}$ (with $\text{dist}(\mathrm{supp}f_{j},\partial \Omega )<2/j$) such that

$$G\left(f_{j}\right)⇀u^{★}\;\;\text{in}\;L_{\text{loc}}^{1}\left(\Omega \right),\;\text{as}\;j↑\infty .$$

This limit function has the boundary behaviour

(1.7) $$u^{★}≍\delta^{(2s-\gamma -1)\wedge \gamma }\;\text{in}\;\Omega ,$$

except in the case γ=s−1/2 when a logarithmic correction is in order.

Note that the exponent is the upper bound of the range in (1.5). We will prove that the problems†

$$\int_{\Omega }u\;\psi =\int_{\partial \Omega }h\;D_{\gamma }\left[G\left(\psi \right)\right]\;\text{for}\;\text{any}\;\psi \in L_{c}^{\infty }\left(\Omega \right).$$

have a unique solution u, which is comparable to $u^{★}$ at the boundary. Going back to the representation in L, this would mean that φ=G[ψ] and then formally

$$\int_{\Omega }u\;L\varphi =\int_{\partial \Omega }h\;D_{\gamma }\left[\varphi \right].$$

Note that this is the formulation of the Dirichlet boundary value problem when L=−Δ so s=γ=1. When $\gamma >s-\frac{1}{2}$, this is even the unique (see Theorems 4.6 and 4.13) weak‐dual solution of problem

(1.8) $$\left\{\begin{array}{cccc} Lu & =0 & & \text{in}\;\Omega ,\\ u & =0 & & \text{in}\;R^{N}\setminus \bar{\Omega }\;\text{(if}\;\text{applicable)},\\ \frac{u}{u^{★}} & =h & & \text{on}\;\partial \Omega . \end{array}\right.$$

#### Comments

Our presentation unifies in a single theory previous results for the RFL ($u^{★}≍\delta^{s-1}$, see [1]), the SFL ($u^{★}≍\delta^{2(s-1)}$, see [2]), the CFL ($u^{★}≍1$, see [14]), and even the classical Laplacian ($u^{★}≍1$).

The case $\gamma <s-\frac{1}{2}\;$, which does not include any of the main known examples, is somewhat particular. In this case, due to (1.7), $0<u^{★}\left(x\right)\to 0$ as x→∂Ω and it is a non‐trivial solution of (1.1) with data f=0. This yields some doubt about the uniqueness of solutions to (1.1)–(1.2). Furthermore, if $\gamma ⩽s-\frac{1}{2}$, then $u^{★}≍\delta^{\gamma }$, which in turn means that the critical solutions have the same boundary behaviour as the solutions for regular data f. This does not seem to be consistent with elliptic problems like (1.1).

### 1.2 Some examples

Large classes of operators L have Green operators G given by (K0)–(K3): here are some notorious examples that are reviewed, for instance, in [3, 10, 40].

#### The restricted fractional Laplacian

The RFL is defined as the singular integral operator

$${\left(-\Delta \right)}_{\mathrm{RFL}}^{s}u\left(x\right)=p.v.\;\int_{R^{n}}\frac{u(x)-u(y)}{{\left|x-y\right|}^{n+2s}}\;dy,$$

up to a multiplicative constant only depending on n and s, and corresponds to the s power of the Laplacian operator defined in $R^{n}$ (which can be equivalently defined via the Fourier transform).

The natural boundary conditions are given in $R^{n}\setminus \bar{\Omega }$

$$\left\{\begin{array}{cc} {\left(-\Delta \right)}_{\mathrm{RFL}}^{s}u=f & \text{in}\;\Omega \\ u=0 & \text{in}\;R^{n}\setminus \bar{\Omega } \end{array}\right.$$

and also have a stochastic interpretation corresponding to killing a Lévy flight upon leaving Ω.

Here we can consider all s∈(0,1) and we have the precise value

γ=s.

Details can be consulted in many references, see, for instance, [11, 37].

#### Perturbations of the RFL

Using the above one, it is possible to build other examples. Here are a couple of interesting operators which are included in our analysis and the corresponding references:

-  ${\left(-\Delta \right)}_{\mathrm{RFL}}^{s}+b\cdot \nabla$ for s∈(1/2,1) and $b\in L^{\infty }\left(\Omega \right)$: in this case (see [9]);
$$\begin{array}{c} \gamma =s. \end{array}$$
-  ${\left(-\Delta \right)}_{\mathrm{RFL}}^{s_{1}}+{\left(-\Delta \right)}_{\mathrm{RFL}}^{s_{2}}$ with $0<s_{2}<s_{1}⩽1$: in this case
$$\begin{array}{ccc} s=\gamma =s_{1} & & \text{for}\;s_{1}<1\;\text{and}\;n>2s_{1},\\ s=\gamma =1 & & \text{for}\;s_{1}=1\;\text{and}\;n⩾3, \end{array}$$
  see, respectively, [16] and [17].

#### The spectral fractional Laplacian

A different way of considering the s power of the Laplacian consists in taking the power of the Dirichlet Laplacian, that is, the Laplacian coupled with homogeneous boundary conditions. This approach typically makes use of an eigenbasis expansion. Let ${\left(\varphi_{m}\right)}_{m\in N}$ be the eigenfunctions of the Laplacian linked to the nondecreasing sequence of eigenvalues $0<\lambda_{1}<\lambda_{2}⩽\text{…}$ (repeated according to their multiplicity)

$$\left\{\begin{array}{cc} -\Delta \varphi_{m}=\lambda_{m}\varphi_{m} & \text{in}\;\Omega ,\\ \varphi_{m}=0 & \text{on}\;\partial \Omega . \end{array}\right.$$

Let $u\in H^{2}\cap H_{0}^{1}\left(\Omega \right)$. Letting ${\hat{u}}_{m}=\int_{\Omega }u\varphi_{m}$, we have the representation

$$-\Delta u=\sum_{m=1}^{+\infty }\lambda_{m}{\hat{u}}_{m}\varphi_{m}.$$

The SFL is the operator with eigenvalues $\lambda_{m}^{s}$ corresponding to eigenfunctions $\varphi_{m}$. Hence, we define

$${\left(-\Delta \right)}_{\mathrm{SFL}}^{s}u=\sum_{m=1}^{+\infty }\lambda_{m}^{s}{\hat{u}}_{m}\varphi_{m}.$$

Since this is an operator‐wise definition we provide the boundary conditions given from the classical operator, and hence the problem is

$$\left\{\begin{array}{cc} {\left(-\Delta \right)}_{\mathrm{SFL}}^{s}u=f & \text{in}\;\Omega ,\\ u=0 & \text{on}\;\partial \Omega . \end{array}\right.$$

We underline how this is not the only possible representation and it is also possible to write it as the singular integral operator

$$\begin{array}{c} {\left(-\Delta \right)}_{\mathrm{SFL}}^{s}u\left(x\right)=p.v.\;\int_{\Omega }\left(u(x)-u(y)\right)\;J\left(x,y\right)\;dt+\kappa \left(x\right)\;u\left(x\right),\;x\in \Omega , \end{array}$$

for

$$\begin{array}{cc} J(x,y) & =\frac{s}{\Gamma (1-s)}\int_{0}^{+\infty }p_{\Omega }\left(t,x,y\right)\;\frac{dt}{t^{1+s}},\\ \kappa (x) & =\frac{s}{\Gamma (1-s)}\int_{0}^{+\infty }\left(1-\int_{\Omega }p_{\Omega }\left(t,x,y\right)\;dy\right)\;\frac{dt}{t^{1+s}}, \end{array}$$

and $p_{\Omega }$ the Dirichlet heat kernel on Ω. It is possible to prove that, when $\partial \Omega \in C^{1,1}$,

(1.9) $$\begin{array}{c} J\left(x,y\right)≍\frac{1}{{\left|x-y\right|}^{n+2s}}\left(\frac{\delta (x)\;\delta (y)}{{\left|x-y\right|}^{2}}\wedge 1\right),\;\kappa \left(x\right)≍\delta {\left(x\right)}^{-2s},\;x,y\in \Omega , \end{array}$$

see [8, Theorem 5.92].

Stochastically speaking, this operator generates a subordinate killed Brownian motion, which is a Brownian motion killed upon hitting ∂Ω and which is then evaluated at random times distributed as an increasing α‐stable process in (0,∞), see [39]. The killing of the Brownian motion as it touches the boundary is encoded in the homogeneous boundary conditions.

Here again s∈(0,1) and in this case,

γ=1.

Details can be consulted in many references, see, for instance, [11, 13].

#### An interpolation of the RFL and the SFL

A family of ‘intermediate’ operators between the RFL and the SFL has been built in [35]. For $\sigma_{1},\sigma_{2}\in \left(0,1\right]$, one can consider the spectral $\sigma_{2}$ power of a RFL of exponent $\sigma_{1}$

$$\begin{array}{c} L_{\sigma_{1},\sigma_{2}}={\left(\;{\left(-\Delta \right)}_{\mathrm{RFL}}^{\sigma_{1}}\;\right)}_{\mathrm{SFL}}^{\sigma_{2}}. \end{array}$$

It is formally clear that

$$\begin{array}{c} L_{\sigma_{1},1}={\left(-\Delta \right)}_{\mathrm{RFL}}^{\sigma_{1}}\;\text{and}\;L_{1,\sigma_{2}}={\left(-\Delta \right)}_{\mathrm{SFL}}^{\sigma_{2}}. \end{array}$$

The Green function associated to this operator satisfies (K2) with

$$\begin{array}{c} s=\sigma_{1}\sigma_{2}\;\text{and}\;\gamma =\sigma_{1}, \end{array}$$

see [35, Theorem 6.4].

#### The censored fractional Laplacian

This operator is defined as

$${\left(-\Delta \right)}_{\mathrm{CFL}}^{s}u\left(x\right)=p.v.\;\int_{\Omega }\frac{u(x)-u(y)}{{\left|x-y\right|}^{n+2s}}\;dy,$$

so that we have identity

$$\begin{array}{c} {\left(-\Delta \right)}_{\mathrm{CFL}}^{s}u={\left(-\Delta \right)}_{\mathrm{RFL}}^{s}u-u\;{\left(-\Delta \right)}_{\mathrm{RFL}}^{s}\chi_{\Omega } \end{array}$$

(recall that the RFL is evaluated only on functions satisfying u=0 in $R^{n}\setminus \bar{\Omega }$).

This operator generates a censored stable process, introduced in [7], a stable process which is confined in Ω and finally killed upon hitting ∂Ω. For this reason, a suitable boundary condition is

$$\begin{array}{c} u=0\;\text{on}\;\partial \Omega . \end{array}$$

Here s∈(1/2,1) and

γ=2s−1,

see [7, 15]. A class of operators which generalises and includes the CFL is covered by the analysis in [18].

## 2 INTERIOR DIRICHLET PROBLEM: EXISTENCE, UNIQUENESS AND INTEGRABILITY

### 2.1 Functional properties of the Green operator

Theorem 2.1 Assume (K0)–(K3). Then G is a continuous operator

(2.1) $$\begin{array}{cc} L^{\infty }\left(\Omega \right)\; & ⟶\;L^{\infty }\left(\Omega \right), \end{array}$$

(2.2) $$\begin{array}{cc} L_{c}^{\infty }\left(\Omega \right)\; & ⟶\;\delta^{\gamma }L^{\infty }\left(\Omega \right), \end{array}$$

(2.3) $$\begin{array}{cc} L^{1}\left(\Omega \right)\; & ⟶\;L^{1}\left(\Omega \right), \end{array}$$

(2.4) $$\begin{array}{cc} L^{1}\left(\Omega ,\delta^{\gamma }\right)\; & ⟶\;L_{\text{loc}}^{1}\left(\Omega \right). \end{array}$$

Moreover, for $f\in L_{c}^{1}\left(\Omega \right)$,

(2.5) $$\left|G\left(f\right)\left(x\right)\right|⩽C\;\delta {\left(x\right)}^{\gamma }\;\mathrm{dist}{\left(x,\mathrm{supp}(f)\right)}^{2s-n-\gamma }\int_{\Omega }\left|f\right|\delta^{\gamma },\;x\in \Omega \setminus \mathrm{supp}\left(f\right).$$

In particular, if $f\in L_{c}^{1}\left(\Omega \right)$, then $\delta^{-\gamma }G\left(f\right)$ is bounded in a neighbourhood of the boundary.

We are going to extensively use assumptions (K0)–(K3) without further notice.

As to (2.1), we simply estimate, for any $f\in L^{\infty }\left(\Omega \right)$ and x∈Ω,

$$\begin{array}{c} |G\left(f\right)\left(x\right)|⩽{∥f∥}_{L^{\infty }\left(\Omega \right)}\int_{\Omega }G\left(x,y\right)\;dy⩽{C∥f∥}_{L^{\infty }\left(\Omega \right)}\int_{\Omega }{\left|x-y\right|}^{2s-n}\;dy⩽C{∥f∥}_{L^{\infty }\left(\Omega \right)}. \end{array}$$

Concerning (2.2), for x∈Ω∖supp(f), we deduce

$$\begin{array}{cc} \left|G(f)(x)\right| & ⩽{∥f∥}_{L^{\infty }\left(\Omega \right)}\int_{\mathrm{supp}(f)}G\left(x,y\right)\;dy\\ & ⩽{C∥f∥}_{L^{\infty }\left(\Omega \right)}\delta {\left(x\right)}^{\gamma }\int_{\mathrm{supp}(f)}{\left|x-y\right|}^{2s-n-2\gamma }\delta {\left(y\right)}^{\gamma }\;dy \end{array}$$

and this proves the result.

For (2.3), we estimate for $f\in L^{1}\left(\Omega \right)$ and using (K1),

$$\begin{array}{c} \int_{\Omega }|G\left(f\right)|⩽\int_{\Omega }\int_{\Omega }G\left(x,y\right)|f\left(y\right)|\;dy\;dx=\int_{\Omega }|f|\;G\left(\chi_{\Omega }\right)⩽C{∥f∥}_{L^{1}\left(\Omega \right)}, \end{array}$$

where we have used (2.1) on $G(\chi_{\Omega })$.

To prove (2.4), we note that, for any K⋐Ω and $f\in L^{1}\left(\Omega ,\delta \right)$, we have

$$\begin{array}{c} \int_{K}|G\left(f\right)|⩽\int_{K}\int_{\Omega }G\left(x,y\right)|f\left(y\right)|\;dy\;dx=\int_{\Omega }|f|\;G\left(\chi_{K}\right)⩽C\int_{\Omega }\left|f\right|\;\delta^{\gamma } \end{array}$$

where we have used (2.2) on $G(\chi_{K})$.

Finally, we prove (2.5). For x∈Ω∖supp(f) we have

$$\begin{array}{cc} \left|G(f)(x)\right| & ⩽\int_{\Omega }G\left(x,y\right)\left|f\left(y\right)\right|\;dy\\ & ⩽C\delta {\left(x\right)}^{\gamma }\int_{\Omega }{|f\left(y\right)|\delta \left(y\right)}^{\gamma }\;dy\;\underset{y\in \mathrm{supp}(f)}{\sup}{\left|x-y\right|}^{2s-n-\gamma }\\ & ⩽C\delta {\left(x\right)}^{\gamma }{∥f\delta^{\gamma }∥}_{L^{1}\left(\Omega \right)}\;\mathrm{dist}{\left(x,\mathrm{supp}(f)\right)}^{2s-n-\gamma } \end{array}$$

and this proves the result.□

Remark 2.2 In Subsection 3.3, we will give a sharper characterisation of the image of map G in terms of weighted $L^{1}$ spaces.

Remark 2.3 Formally, one could take μ∈M(Ω) and estimate

(2.6) $$\begin{array}{c} \int_{\Omega }|G\left(\mu \right)|⩽\int_{\Omega }\int_{\Omega }G\left(x,y\right)\;d|\mu |\left(y\right)\;dx=\int_{\Omega }G\left(\chi_{\Omega }\right)\;d|\mu |⩽C|\mu |\left(\Omega \right) \end{array}$$

where we have used (2.1) on $G(\chi_{\Omega })$ or take $\mu \in M(\Omega ,\delta^{\gamma })$ and estimate, for any K⋐Ω

(2.7) $$\begin{array}{c} \int_{K}|G\left(\mu \right)|⩽\int_{K}\int_{\Omega }G\left(x,y\right)\;d|\mu |\left(y\right)\;dx=\int_{\Omega }G\left(\chi_{K}\right)\;d|\mu |⩽C\int_{\Omega }\delta^{\gamma }d\left|\mu \right| \end{array}$$

where we have used (2.2) on $G(\chi_{K})$. This computation is justified in the typical examples where G is continuous. However, since we have made no continuity assumptions for G, it is possible that integration against a measure is not defined. We will give more details on this case in Subsection 2.5.

### 2.2 Weak‐dual formulation

If L is self‐adjoint, equations of type (1.1) are typically written in *very weak* form as

(2.8) $$\int_{\Omega }u\;L\varphi =\int_{\Omega }f\varphi$$

for all test functions φ in some adequate space given by the operator and the boundary conditions. Since we want to tackle multiple types of operators and boundary conditions, we focus instead on the *weak‐dual* formulation (see, for example, [10]). This is formulated instead in terms of the inverse operator G, which is taken as an *a priori*. This allows to avoid giving a meaning to Lφ.

Definition 2.4 Given $f\in L^{1}\left(\Omega ,\delta^{\gamma }\right)$, a function $u\in L_{\text{loc}}^{1}\left(\Omega \right)$ is a weak‐dual solution of problem (1.1) if

(2.9) $$\int_{\Omega }u\psi =\int_{\Omega }f\;G\left(\psi \right),\;\;\text{for}\;\text{any}\;\psi \in L_{c}^{\infty }\left(\Omega \right).$$

Note that this weak‐dual formulation is equivalent to take test functions $\varphi \in G(L_{c}^{\infty }\left(\Omega \right))$ in (2.8). Also, we underline how the integral in the right‐hand side of (2.9) is finite in view of (2.2).

Theorem 2.5 Assume (K0)–(K3) and let $f\in L^{1}\left(\Omega ,\delta^{\gamma }\right)$. Then, there exists a unique function $u\in L_{\text{loc}}^{1}\left(\Omega \right)$ satisfying

(2.10) $$\int_{\Omega }u\psi =\int_{\Omega }f\;G\left(\psi \right)\;\text{for}\;\text{any}\;\psi \in L_{c}^{\infty }\left(\Omega \right).$$

This function is precisely u=G(f) and it satisfies

$$\int_{K}|u|⩽∥f\delta^{\gamma }{∥}_{L^{1}\left(\Omega \right)}{\left\|\frac{G(\chi_{K})}{\delta^{\gamma }}\right\|}_{L^{\infty }\left(\Omega \right)}\;\text{for}\;\text{any}\;K⋐\Omega .$$

Let us first note that $u=G\left(f\right)\in L_{\text{loc}}^{1}\left(\Omega \right)$ in view of (2.4). It formally satisfies (2.10) as a consequence of (K1) by the Fubini's theorem. This formal bounds are indeed rigourous for $f\in L_{c}^{\infty }\left(\Omega \right)$. Furthermore, due to the bounds provided by Theorem 2.1 one can pass to the limit in approximations.

We now focus on uniqueness. Let $u_{1},u_{2}$ be two solutions to (2.10). Then

$$\int_{\Omega }\left(u_{1}-u_{2}\right)\;\psi =0,\;\text{for}\;\text{any}\;\psi \in L_{c}^{\infty }\left(\Omega \right).$$

Let K⋐Ω and $\psi =\mathrm{sign}\left(u_{1}-u_{2}\right)\chi_{K}\in L_{c}^{\infty }\left(\Omega \right)$. Using this as a test function, we deduce

$$\int_{K}\left|u_{1}-u_{2}\right|=0.$$

Since this holds for every K⋐Ω, we have that $u_{1}=u_{2}$ a.e. in Ω. Also, we have that

$$\int_{K}\left|u\right|⩽\int_{K}|G\left(f\right)|⩽\int_{\Omega }|f|\;G\left(\chi_{K}\right)⩽{∥f\delta^{\gamma }∥}_{L^{1}\left(\Omega \right)}{\left\|\frac{G(\chi_{K})}{\delta^{\gamma }}\right\|}_{L^{\infty }\left(\Omega \right)}$$

which is a nontrivial inequality thanks to (2.2).□

### 2.3 Optimal class of data and a lower Hopf estimate

Theorem 2.6 (Lower Hopf) Assume (K0)–(K3). There exists c>0 such that, for all f⩾0,

$$G\left(f\right)\left(x\right)⩾c\;\delta {\left(x\right)}^{\gamma }\int_{\Omega }f\left(y\right)\;\delta {\left(y\right)}^{\gamma }\;dy,\;x\in \Omega .$$

By assumption (K0), it is sufficient to prove that

(2.11) $$G\left(x,y\right)⩾c{\left(\delta (x)\delta (y)\right)}^{\gamma },\;x,y\in \Omega .$$

Assume, towards a contradiction, this is not true. Then, there exist sequences of points ${\left(x_{j}\right)}_{j\in N},{\left(y_{j}\right)}_{j\in N}\subseteq \Omega$ such that

$$\frac{G(x_{j},y_{j})}{\delta {\left(x_{j}\right)}^{\gamma }\delta {\left(y_{j}\right)}^{\gamma }}\to 0,\;\text{as}\;j↑\infty .$$

By assumption (K2), either

$$|x_{j}-y_{j}{|}^{2s-n-2\gamma }\to 0,\;\text{as}\;j↑\infty ,$$

which is not possible since Ω is bounded and 2s−n−2γ⩽0 (cf. (K3)), or

$$\frac{|x_{j}-y_{j}{|}^{2s-n}}{\delta {\left(x_{j}\right)}^{\gamma }\delta {\left(y_{j}\right)}^{\gamma }}\to 0,\;\text{as}\;j↑\infty .$$

Since Ω is bounded, δ is bounded, and hence we should have that $|x_{j}-y_{j}{|}^{2s-n}\to 0$ as j↑∞ (cf. (K3)). Again, this is not possible. We arrive to a contradiction and (2.11) is proven.□

Corollary 2.7 Assume (K0)–(K3) and let K⋐Ω. Then

$$G\left(\chi_{K}\right)\left(x\right)≍\delta {\left(x\right)}^{\gamma },\;x\in \Omega .$$

It follows from Theorem 2.6 and (2.2).□

Remark 2.8 If $0⩽f\notin L^{1}\left(\Omega ,\delta^{\gamma }\right)$ and $f_{k}=f\wedge k,\;k\in N$, then, for every x∈Ω,

$$G\left(f_{k}\right)\left(x\right)⩾c\delta {\left(x\right)}^{\gamma }\int_{\Omega }f_{k}\left(y\right)\delta {\left(y\right)}^{\gamma }\;dy\to +\infty ,\;\text{as}\;k↑+\infty ,$$

due to the monotone convergence theorem.

Thanks to Theorem 2.6 and Remark 2.8, we have shown that $L^{1}\left(\Omega ,\delta^{\gamma }\right)$ is the *optimal class of data*.

### 2.4 Uniform integrability over compacts

Let us show that G maps $L^{1}$‐bounded sequences into $L^{1}$‐weakly pre‐compact sequences.

Lemma 2.9 Assume (K0)–(K3) and let $f\in L^{1}\left(\Omega ,\delta^{\gamma }\right)$, K⋐Ω. Then, for A⊂K,

$$\int_{A}\left|G\left(f\right)\right|⩽C_{K,\beta }{\left|A\right|}^{\beta }{∥f\delta^{\gamma }∥}_{L^{1}\left(\Omega \right)},\;\;\text{for}\;\text{any}\;0<\beta <\frac{2s}{n}.$$

In particular, for any K⋐Ω, G maps bounded sequences in $L^{1}\left(\Omega ,\delta^{\gamma }\right)$ into uniformly integrable sequences in K.

We have that

$$\begin{array}{cc} \int_{A}\left|G\left(f\right)\right| & ⩽\int_{A}\int_{\Omega }G\left(x,y\right)f\left(y\right)\;dy\;dx=\int_{\Omega }\left|f\left(y\right)\right|\left(\int_{A}G\left(x,y\right)dx\right)dy\\ & =\int_{\Omega }\left|f\left(y\right)\right|\delta {\left(y\right)}^{\gamma }\left(\int_{A}\frac{G(x,y)}{\delta {\left(y\right)}^{\gamma }}\;dx\right)dy \end{array}$$

We take $1<p<\frac{n}{n-2s}$. Due to the Hölder's inequality,

$$\int_{A}\frac{G(x,y)}{\delta {\left(y\right)}^{\gamma }}\;dx⩽{\left|A\right|}^{\frac{1}{p^{\prime }}}{\left(\int_{K}{\left|\frac{G(x,y)}{\delta {\left(y\right)}^{\gamma }}\right|}^{p}dx\right)}^{\frac{1}{p}},\;\;p^{\prime }=\frac{p}{p-1}>\frac{n}{2s}.$$

We estimate this last integral to recover $C_{K}$. For any y∈Ω such that dist(y,K)<dist(K,∂Ω)/2 we have that

$$\delta \left(y\right)=\mathrm{dist}\left(y,\partial \Omega \right)>\mathrm{dist}\left(K,\partial \Omega \right)-\mathrm{dist}\left(y,K\right)>\frac{1}{2}\mathrm{dist}\left(K,\partial \Omega \right)$$

and hence

$$\begin{array}{ccc} \int_{K}{\left|\frac{G(x,y)}{\delta {\left(y\right)}^{\gamma }}\right|}^{p}dx & ⩽ & {\left(\frac{\mathrm{dist}(K,\partial \Omega )}{2}\right)}^{-\gamma p}\int_{K}G{\left(x,y\right)}^{p}dy\\ & ⩽ & C{\left(\frac{\mathrm{dist}(K,\partial \Omega )}{2}\right)}^{-\gamma p}\int_{K}{\left|x-y\right|}^{-p(n-2s)}dx⩽C{\left(\frac{\mathrm{dist}(K,\partial \Omega )}{2}\right)}^{-\gamma p} \end{array}$$

since p(n−2s)<n, where C depends only on p and Ω. One the other hand, if y is such that dist(y,K)⩾dist(K,∂Ω)/2, for x∈K we have |x−y|>dist(y,K) and so we compute

$$\begin{array}{ccc} \int_{K}{\left|\frac{G(x,y)}{\delta {\left(y\right)}^{\gamma }}\right|}^{p}dx & ⩽ & C\int_{K}\frac{\delta {\left(x\right)}^{\gamma p}}{{\left|x-y\right|}^{p(n-2s+2\gamma )}}dx\\ & ⩽ & C{\left(\frac{\mathrm{dist}(K,\partial \Omega )}{2}\right)}^{p(-n+2s-2\gamma )}\int_{K}\delta {\left(x\right)}^{\gamma p}dx⩽C{\left(\frac{\mathrm{dist}(K,\partial \Omega )}{2}\right)}^{p(-n+2s-2\gamma )}, \end{array}$$

where C depends only on p and Ω. This completes the proof.□

### 2.5 Measure data and continuous solutions

Under mild assumptions on the Green kernel G, it is possible to improve (2.1) and (2.2) to higher regularity of solutions. By duality, this allows more general data in (1.1) and we are particularly interested in measure data. For this reason, let us assume that

(K4) $$\begin{array}{c} \text{For}\;\text{any}\;\text{sequence}\;\Omega ∋x_{j}\to x\;\text{as}\;j↑\infty \;\text{we}\;\text{have}\;\lim_{j↑\infty }G\left(x_{j},\cdot \right)=G\left(x,\cdot \right)\;\text{a.e.}\;\text{in}\;\Omega . \end{array}$$

Theorem 2.10 Assume (K0)–(K4). Then the operator G maps

$$\begin{array}{cc} L^{\infty }\left(\Omega \right) & \;⟶\;C(\bar{\Omega })\\ L_{c}^{\infty }\left(\Omega \right) & \;⟶\;\delta^{\gamma }C\left(\bar{\Omega }\right). \end{array}$$

In view of (2.1) and (2.2), we just need to justify the continuity claim. Let us consider $f\in L^{\infty }\left(\Omega \right)$. To prove continuity we select an x∈Ω, and ${\left(x_{j}\right)}_{j\in N}\subset \bar{\Omega }\;\text{such}\;\text{that}\;x_{j}\to x\;\text{as}\;j↑\infty$. By assumption (K4) we know that $G\left(x_{j},\cdot \right)\to G\left(x,\cdot \right)$ a.e. in Ω. Moreover, let us note that ${\left(G\left(x_{j},\cdot \right)\right)}_{j\in N}\subset L^{p}\left(\Omega \right)$, p∈[1,n/(n−2s)) is uniformly bounded, since

$$\begin{array}{c} \int_{\Omega }{G(x_{j},y)}^{p}\;dy⩽\int_{\Omega }\frac{dy}{{|x_{j}-y|}^{(n-2s)p}} \end{array}$$

and Ω is bounded. Therefore, so is ${\left(G\left(x_{j},\cdot \right)f\right)}_{j\in N}$. Due to the weak compactness in reflexive spaces it is convergent. Applying (K4) we can compute the pointwise limit

$$\begin{array}{c} G\left(f\right)\left(x_{j}\right)=\int_{\Omega }G\left(x_{j},y\right)\;f\left(y\right)\;dy⟶\int_{\Omega }G\left(x,y\right)\;f\left(y\right)\;dy=G\left(f\right)\left(x\right)\;\text{as}\;j↑\infty . \end{array}$$

This proves that $G\left(f\right)\in C\left(\bar{\Omega }\right)$.

Let $f\in L_{c}^{\infty }\left(\Omega \right)$. Since we have already proven that G(f)∈C(Ω), we only need to prove that $\delta^{-\gamma }G\left(f\right)$ is continuous on some small neighbourhood of ∂Ω. Consider ε>0 small enough so that K=supp(f)⊂{δ⩾2ε}⋐Ω. Let U={δ<ε} be the small neighbourhood of ∂Ω. We have that

$$\begin{array}{c} \int_{K}\frac{{G(x,y)}^{p}}{\delta {\left(x_{j}\right)}^{\gamma p}}\;dy⩽\int_{K}\frac{{\delta (y)}^{\gamma p}}{{\left|x-y\right|}^{(n-2s+2\gamma )p}}dy⩽C_{\varepsilon },\;\forall x\in U. \end{array}$$

Select now an x∈U and let $\Omega \in x_{j}\to x$ as j↑+∞. Since U is open, then $x_{j}\in U$ for j large enough. Again, by weak compactness,

$$\delta {\left(x_{j}\right)}^{-\gamma }G\left(f\right)\left(x_{j}\right)=\int_{K}\frac{G(x_{j},y)}{{\delta (x_{j})}^{\gamma }}\;f\left(y\right)\;dy⟶\int_{K}\frac{G(x,y)}{{\delta (x)}^{\gamma }}\;f\left(y\right)\;dy=\delta {\left(x\right)}^{-\gamma }G\left(f\right)\left(x\right)\;\text{as}\;j↑\infty .$$

This completes the proof.□

With this new machinery, we can justify the intuition given by Remark 2.3.

Theorem 2.11 Assume (K0)–(K4). Then, G maps

$$\begin{array}{cc} M(\Omega )\; & ⟶\;L^{1}\left(\Omega \right)\\ M(\Omega ,\delta^{\gamma })\; & ⟶\;L_{\text{loc}}^{1}\left(\Omega \right). \end{array}$$

Furthermore, for every $\mu \in M(\Omega ,\delta^{\gamma })$, u=G(μ) is the unique $u\in L_{\text{loc}}^{1}\left(\Omega \right)$ such that

(2.12) $$\int_{\Omega }u\psi =\int_{\Omega }G\left(\psi \right)\;d\mu \;\text{for}\;\text{any}\;\psi \in L_{c}^{\infty }\left(\Omega \right).$$

holds. Moreover, it satisfies

$$\int_{K}\left|u\right|⩽\left(\int_{\Omega }\delta^{\gamma }d\left|\mu \right|\right){\left\|\frac{G(\chi_{K})}{\delta^{\gamma }}\right\|}_{L^{\infty }\left(\Omega \right)}\;\text{for}\;\text{any}\;K⋐\Omega .$$

Due to (K4), G(μ) is now a well‐defined integral. Now we can apply (2.6) and (2.7). Moreover, also $\int_{\Omega }G\left(\mu \right)\;\psi$ is well‐defined for any $\psi \in L_{c}^{\infty }\left(\Omega \right)$. Note that, in view of (2.2), we have

$$\begin{array}{c} \int_{\Omega }\int_{\Omega }G\left(x,y\right)\;|\psi \left(x\right)|\;dx\;d|\mu |\left(y\right)⩽C_{\psi }\int_{\Omega }\delta {\left(y\right)}^{\gamma }\;d\left|\mu \right|\left(y\right)<+\infty , \end{array}$$

so that we can apply the Fubini's theorem and (K1) to deduce

$$\begin{array}{c} \int_{\Omega }G\left(\mu \right)\;\psi =\int_{\Omega }G\left(\psi \right)\;d\mu \end{array}$$

which proves (2.12). We now show uniqueness. Let $u_{1},u_{2}$ be two solutions to (2.12). Then

$$\int_{\Omega }\left(u_{1}-u_{2}\right)\;\psi =0,\;\text{for}\;\text{any}\;\psi \in L_{c}^{\infty }\left(\Omega \right).$$

Let K⋐Ω and $\psi =\mathrm{sign}\left(u_{1}-u_{2}\right)\chi_{K}\in L_{c}^{\infty }\left(\Omega \right)$. Using this as a test function, we deduce

$$\int_{K}\left|u_{1}-u_{2}\right|=0.$$

Since this holds for every K⋐Ω, we have that $u_{1}=u_{2}$ a.e. in Ω. Also, we have that

$$\int_{K}\left|u\right|⩽\int_{K}|G\left(\mu \right)|⩽\int_{\Omega }G\left(\chi_{K}\right)\;d\left|\mu \right|⩽\left(\int_{\Omega }\delta^{\gamma }d\left|\mu \right|\right){\left\|\frac{G(\chi_{K})}{\delta^{\gamma }}\right\|}_{L^{\infty }\left(\Omega \right)}$$

which is a nontrivial inequality thanks to (2.2).□

## 3 BREAKDOWN OF THE BOUNDARY CONDITION IN THE INTERIOR PROBLEM

We address now the main question of this paper, which is the violation of the boundary data in the optimal theory for the interior problem. We give precise answers of the anomalous boundary behaviour in terms of the behaviour of the forcing data.

### 3.1 Range of exponents

Before stating and proving the main result of this paragraph, we need to state a couple of technical estimates on which the result is based. Since the proofs of these estimates is rather long and technical, we defer them to Appendix B. The first one gives some interior estimates; the second one is describing the sharp behaviour of solutions at the boundary.

Remark 3.1 In what follows, we use without further note ε>0 to denote the fixed width on which the tubular neighbourhood theorem can be rightfully applied, that is, the map

$$\begin{array}{ccc} \Phi :\partial \Omega \times (-\varepsilon ,\varepsilon ) & ⟶ & R^{n}\\ \left(z,\delta \right) & \mapsto & z+\delta n(z) \end{array}$$

defines a diffeomorphism to its image. Here, n represents the interior unit normal. This is well‐known for smooth manifolds (see [21]), and holds also for $C^{1,1}$ open sets of $R^{n}$. The notation δ might seem like an abuse of notation, but it will lead to no confusion since, in this setting, dist(Φ(z,δ),∂Ω)=δ for ε sufficiently small.

Lemma 3.2 Assume that (K0)–(K3) hold. Moreover, assume β+γ>−1 and let η<ε be fixed. Then there exists a constant $\bar{c}\left(\eta \right)>0$ such that, for any x∈{δ>η/2}, it holds

(3.1) $$\begin{array}{c} G\left(\delta^{\beta }\chi_{\left\{\delta <\eta \right\}}\right)\left(x\right)⩽\bar{c}\left(\eta \right). \end{array}$$

Moreover, if $\gamma <s-\frac{1}{2}$ and x∈{δ>η/2}, then

(3.2) $$\begin{array}{c} G\left(\delta^{\beta }\chi_{\left\{\delta <\eta \right\}}\right)\left(x\right)⩽\eta^{\beta +\gamma +1}\delta {\left(x\right)}^{\gamma } \end{array}$$

up to constants not depending on η.

**FIGURE 3:**
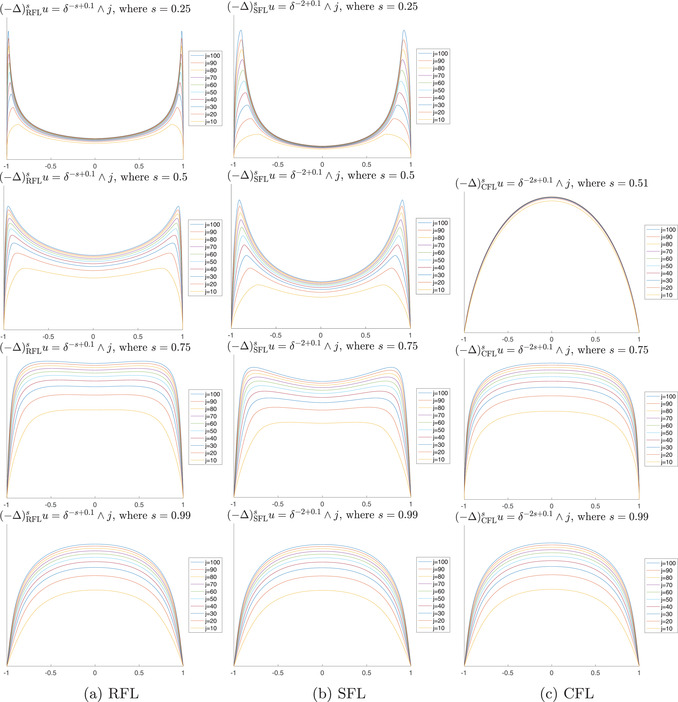
Numerical solutions of $Lu=\delta^{-\gamma -1+\varepsilon }\wedge j\;$ in dimension n=1 for different operators. All computations correspond to Finite Difference numerical schemes. For the RFL we take the weights for the finite difference discretisation of the fractional Laplacian in $R^{n}$ in [22] (see also [19]). The discretisation for smooth functions is rigorously shown to be $O(h^{2})$. A previous approach by Finite Differences is given in [34]. Experimental results in [34] suggest that the restriction for RFL is of order $O(h^{s})$. For the SFL we use as a discretisation the fractional power of the finite differences matrix of the usual Laplacian (−Δ): it is known that the eigenvalues of this matrix converge to those of the usual Laplacian, and hence its fractional power produces a convergent scheme for the SFL. A different scheme can be found in [20]. For the CFL we have used a novel approach, which we will describe in an upcoming paper.

In Figure 3 we have included numerical simulations of $Lu=\delta^{\gamma -1+0.1}\wedge j$ for different examples of L

Lemma 3.3 Assume that (K0)–(K3) hold. Moreover, assume β+γ>−1 and let† η<ε be fixed. Then for any x∈{δ<η/2} it holds

(3.3) $$\begin{array}{c} G\left(\delta^{\beta }\chi_{\left\{\delta <\eta \right\}}\right)\left(x\right)≍\delta {\left(x\right)}^{\beta +2s}+\eta^{\beta +\gamma +1}\delta {\left(x\right)}^{\gamma }+\Theta \left(\eta ,x\right), \end{array}$$

where the Θ is defined as follows:

(a) if $\gamma <s-\frac{1}{2}$, then
(3.4) $$\begin{array}{c} \Theta \left(\eta ,x\right):=\delta {\left(x\right)}^{\beta +2\gamma +1}; \end{array}$$
(b) if $\gamma =s-\frac{1}{2}$, then
(3.5) $$\begin{array}{c} \Theta \left(\eta ,x\right):=\delta {\left(x\right)}^{\beta +2s}\;|\;\ln\delta \left(x\right)|+\eta^{\beta +\gamma +1}\;\left|\;\ln\eta \right|\;\delta {\left(x\right)}^{\gamma }; \end{array}$$
(c) if $\gamma >s-\frac{1}{2}$, then
(3.6) $$\begin{array}{c} \Theta \left(\eta ,x\right):=\left\{\begin{array}{cccc} & 0 & & \text{if}\;\beta <\gamma -2s,\\ & \delta {\left(x\right)}^{\beta +2s}\;\left|\;\ln\left(\delta \left(x\right)/\eta \right)\right| & & \text{if}\;\beta =\gamma -2s,\\ & \eta^{\beta +2s-\gamma }\delta {\left(x\right)}^{\gamma } & & \text{if}\;\beta >\gamma -2s. \end{array}\right. \end{array}$$

We are now ready to prove the following estimate.

Theorem 3.4 Assume that (K0)–(K3) hold. Moreover, assume β+γ>−1. Then $\delta^{\beta }\in L^{1}\left(\Omega ,\delta^{\gamma }\right)$ and

$$G\left(\delta^{\beta }\right)≍\delta^{\alpha }$$

with

(3.7) $$\alpha =\left\{\begin{array}{cccc} & \gamma & & \text{if}\;\beta >\gamma -2s\\ & \gamma \;\text{(and}\;\text{log.}\;\text{weight)}\; & & \text{if}\;\beta =\gamma -2s\;\text{and}\;\gamma >s-\frac{1}{2}\\ & \beta +2s & & \text{if}\;\beta <\gamma -2s\;\text{and}\;\gamma >s-\frac{1}{2}, \end{array}\right.$$

where by logarithmic weight we mean that $G\left(\delta^{\gamma -2s}\right)≍\delta^{\gamma }\;\left(1+|\;\ln\delta |\right)$.

Equation (3.7) can be interpreted by means of formula (1.5) and Figure 1.

Let us first note that conditions β⩽γ−2s and $\gamma ⩽s-\frac{1}{2}$ are not compatible: indeed, if they both held, then it would be β+γ⩽2γ−2s⩽−1 contradicting our standing assumption on β.

We pick some fixed η<ε and we write

$$G\left(\delta^{\beta }\right)=G\left(\delta^{\beta }\chi_{\left\{\delta ⩽\eta \right\}}\right)+G\left(\delta^{\beta }\chi_{\left\{\delta >\eta \right\}}\right).$$

Note that

$$\begin{array}{c} G\left(\delta^{\beta }\chi_{\left\{\delta >\eta \right\}}\right)≍\delta^{\gamma } \end{array}$$

as $\delta^{\beta }\chi_{\left\{\delta >\eta \right\}}\in L_{c}^{\infty }\left(\Omega \right)$. For the other term, we exploit Lemma 3.3 and (3.1) to say

$$\begin{array}{c} G\left(\delta^{\beta }\chi_{\left\{\delta ⩽\eta \right\}}\right)≍\delta {\left(x\right)}^{\beta +2s}+\delta {\left(x\right)}^{\gamma }+\Theta \left(1,x\right), \end{array}$$

where Θ is defined as in Lemma 3.3. Now, the asymptotic behaviour is driven by the least exponent on δ, yielding the situation depicted in (3.7).□

Remark 3.5 Let us look at the ranges for α and β as in Theorem 3.4, disregarding the logarithmic cases, to better understand the possible boundary behaviours of solutions to (1.1). When $\gamma >s-\frac{1}{2}$ the admissible range for β is (−1−γ,+∞); in this case α runs in (2s−γ−1,γ]: note that 2s−γ−1 might be negative, meaning that also α is allowed to be negative in some cases. This translates in particular into a rebuttal of $G(\delta^{\beta })=0$ on ∂Ω, despite the fact that this would be the solution to a homogeneous boundary (or exterior problem) value problem. For exterior problem, this shows solutions are discontinuous on the boundary for some singular data (possibly outside $L^{1}\left(\Omega \right)$). For boundary value problems, this is a breakdown of the boundary condition. However, this behaviour intrinsic to the problem, since we are only constructing the closure of the solution operator G, to its maximal domain of definition.

If instead $\gamma <s-\frac{1}{2}$, then again β ranges in (−γ−1,+∞), but this time α is bound to be equal to γ, meaning that there is no range for α.

Example 3.6 Let us exemplify the statement of Theorem 3.4. If we consider β=0, we deduce

$$\begin{array}{c} G\left(\chi_{\Omega }\right)\;≍\;\left\{\begin{array}{cccc} & \delta^{\gamma } & & \text{if}\;\gamma <2s\\ & \delta^{\gamma }\;\left(1+|\;\ln\delta |\right) & & \text{if}\;\gamma =2s\\ & \delta^{2s} & & \text{if}\;\gamma >2s \end{array}\right.\;\text{in}\;\Omega . \end{array}$$

Setting β=γ gives

$$\begin{array}{c} G\left(\delta^{\gamma }\right)≍\delta^{\gamma },\;\text{in}\;\Omega . \end{array}$$

Taking β=±s returns, respectively,

$$\begin{array}{c} G\left(\delta^{s}\right)\;≍\;\left\{\begin{array}{cccc} & \delta^{\gamma } & & \text{if}\;\gamma <3s\\ & \delta^{\gamma }\;\left(1+|\;\ln\delta |\right) & & \text{if}\;\gamma =3s\\ & \delta^{3s} & & \text{if}\;\gamma >3s \end{array}\right.\;\text{and}\;G\left(\delta^{-s}\right)\;≍\;\left\{\begin{array}{cccc} & \delta^{\gamma } & & \text{if}\;\gamma <s\\ & \delta^{\gamma }\;\left(1+|\;\ln\delta |\right) & & \text{if}\;\gamma =s\\ & \delta^{s} & & \text{if}\;\gamma >s. \end{array}\right. \end{array}$$

The value β=−2s is a somewhat critical value for the boundary behaviour (if γ>2s−1, otherwise G is not defined), since

$$\begin{array}{c} G(\delta^{-2s})\;≍\;1. \end{array}$$

Below this value, if β is of the form β=−2s−ε,ε∈(0,γ−2s+1)∖{−γ}, one has

$$\begin{array}{c} G\left(\delta^{-2s-\varepsilon }\right)\;≍\;\delta^{-\varepsilon }. \end{array}$$

Remark 3.7 Note that, if β∈(−1/2,−2s) we have that $\delta^{\beta }\in L^{2}\left(\Omega \right)$ and $G\left(\delta^{\beta }\right)\notin L^{\infty }\left(\Omega \right)$. This is possible if s∈(0,1/4). Hence, this breakdown of the boundary conditions happens *inside* the variational (energy) theory. This should not be surprising since, for s<1/2, $H_{0}^{s}=H^{s}$ (the space has no trace). This points to an essential difference between the properties of the classical Laplacian and the fractional Laplacian for small values of s.

### 3.2 Subcritical boundary behaviour in average terms

We have an extension of the result for the classical Laplacian on averaged convergence to the boundary, see [36].

Lemma 3.8 Assume that (K0)–(K3) hold and let $f\in L^{1}\left(\Omega ,\delta^{\gamma }\right)$.

(a) If γ>s−1/2,
$$\frac{1}{\eta }\int_{\left\{\delta <\eta \right\}}\frac{\left|G(f)\right|}{\delta^{2s-\gamma -1}}⟶0\;\text{as}\;\eta ↓0.$$
(b) If γ=s−1/2,
$$\frac{1}{\eta \;|\;\ln\eta |}\int_{\left\{\delta <\eta \right\}}\frac{\left|G(f)\right|}{\delta^{\gamma }}⟶0\;\text{as}\;\eta ↓0.$$
(c) If γ<s−1/2,
$$\frac{1}{\eta }\int_{\left\{\delta <\eta \right\}}\frac{\left|G(f)\right|}{\delta^{\gamma }}⩽C.$$

Assume that f⩾0. Let us start from (a). It is clear that, by duality,

$$\eta^{-1}\int_{\left\{\delta <\eta \right\}}\frac{G(f)}{\delta^{2s-\gamma -1}}=\int_{\Omega }f\delta^{\gamma }\;\frac{G(\delta^{-2s+\gamma +1}\chi_{\left\{\delta <\eta \right\}})}{\eta \delta^{\gamma }}.$$

We decompose this last integral into two

$$\int_{\Omega }f\delta^{\gamma }\frac{G(\delta^{-2s+\gamma +1}\chi_{\left\{\delta <\eta \right\}})}{\eta \delta^{\gamma }}=\int_{\left\{\delta ⩽\eta /2\right\}}f\delta^{\gamma }\frac{G(\delta^{-2s+\gamma +1}\chi_{\left\{\delta <\eta \right\}})}{\eta \delta^{\gamma }}+\int_{\left\{\delta >\eta /2\right\}}f\delta^{\gamma }\;\frac{G(\delta^{-2s+\gamma +1}\chi_{\left\{\delta <\eta \right\}})}{\eta \delta^{\gamma }}.$$

Using (3.3) and (3.6), in {δ<η/2} we get

$$\begin{array}{c} \frac{G(\delta^{-2s+\gamma +1}\chi_{\left\{\delta <\eta \right\}})}{\eta \delta^{\gamma }}≍\frac{\delta }{\eta }+\eta^{-2s+2\gamma +1}+1⩽3 \end{array}$$

and therefore

$$\begin{array}{c} \int_{\left\{\delta ⩽\eta /2\right\}}f\delta^{\gamma }\frac{G(\delta^{-2s+\gamma +1}\chi_{\left\{\delta <\eta \right\}})}{\eta \delta^{\gamma }}⟶0\;\text{as}\;\eta ↓0 \end{array}$$

by dominated convergence. On the other hand, in {δ>η/2} we have, for σ∈(0,2γ−2s+1),

$$\begin{array}{c} G\left(\delta^{-2s+\gamma +1}\chi_{\left\{\delta <\eta \right\}}\right)⩽\eta^{1+\sigma }G\left(\delta^{-2s+\gamma -\sigma }\chi_{\left\{\delta <\eta \right\}}\right)⩽\eta^{1+\sigma }G\left(\delta^{-2s+\gamma -\sigma }\right)≍\eta^{1+\sigma }\delta^{\gamma -\sigma } \end{array}$$

where we have used Theorem 3.4. As a consequence

$$\begin{array}{c} \int_{\left\{\delta >\eta /2\right\}}f\delta^{\gamma }\frac{G(\delta^{-2s+\gamma +1}\chi_{\left\{\delta <\eta \right\}})}{\eta \delta^{\gamma }}⩽\eta^{\sigma }\int_{\left\{\delta >\eta /2\right\}}f\delta^{\gamma -\sigma }⟶0 \end{array}$$

again by dominated convergence.

The proof of (b) is analogous by using (3.5).

Let us now consider (c). As above, by duality,

$$\eta^{-1}\;\int_{\left\{\delta <\eta \right\}}\frac{G(f)}{\delta^{\gamma }}=\;\int_{\Omega }f\delta^{\gamma }\;\frac{G(\delta^{-\gamma }\chi_{\left\{\delta <\eta \right\}})}{\eta \;\delta^{\gamma }}=\;\int_{\left\{\delta >\eta /2\right\}}f\delta^{\gamma }\;\frac{G(\delta^{-\gamma }\chi_{\left\{\delta <\eta \right\}})}{\eta \;\delta^{\gamma }}+\;\int_{\left\{\delta <\eta /2\right\}}f\delta^{\gamma }\;\frac{G(\delta^{-\gamma }\chi_{\left\{\delta <\eta \right\}})}{\eta \;\delta^{\gamma }}.$$

For the first integral, we use (3.2) with β=−γ to deduce

$$\begin{array}{c} \int_{\left\{\delta >\eta /2\right\}}f\delta^{\gamma }\;\frac{G(\delta^{-\gamma }\chi_{\left\{\delta <\eta \right\}})}{\eta \;\delta^{\gamma }}⩽\int_{\left\{\delta >\eta /2\right\}}f\;\delta^{\gamma } \end{array}$$

up to constants not depending on η. For the second one we use (3.3) and (3.4) which give

$$\begin{array}{c} \frac{G(\delta^{-\gamma }\chi_{\left\{\delta <\eta \right\}})}{\eta \;\delta^{\gamma }}≍\frac{\delta^{-2\gamma +2s}}{\eta }+1+\frac{\delta (x)}{\eta }⩽3,\;\text{in}\;\left\{\delta <\eta \right\}, \end{array}$$

and therefore

$$\begin{array}{c} \int_{\left\{\delta <\eta /2\right\}}f\delta^{\gamma }\;\frac{G(\delta^{-\gamma }\chi_{\left\{\delta <\eta \right\}})}{\eta \;\delta^{\gamma }}⩽C\int_{\Omega }\left|f\right|\;\delta^{\gamma }. \end{array}$$

This completes the proof for f⩾0.

If f changes sign, then can we apply the result have to $f_{+}$ and $f_{-}$.□

### 3.3 Sharp weighted spaces for the Green operator

The computations above allow to complement the analysis carried out in Theorem 2.1, and improve the estimate for the optimal data from $L_{\text{loc}}^{1}$ to a weighted space. It follows the general philosophy that, due to (2.10), for any $\mu \in M(\Omega ,\delta^{\gamma })$ we have

$$G:L^{1}\left(\Omega ,G(\mu )\right)\to L^{1}\left(\Omega ,\mu \right).$$

The result is as follows.

Theorem 3.9 Assume that (K0)–(K3) hold and let α>(γ−2s)∨(−γ−1). We have that

$$G:L^{1}\left(\Omega ,\delta^{\gamma }\right)\to L^{1}\left(\Omega ,\delta^{\alpha }\right)$$

is well‐defined and continuous.

Take $f\in L^{1}\left(\Omega ,\delta^{\gamma }\right)$. Then

$$\begin{array}{c} \int_{\Omega }|G\left(f\right)|\delta^{\alpha }⩽\int_{\Omega }\left|f\right|\;G\left(\delta^{\alpha }\right). \end{array}$$

As α>−γ−1 by assumption, we can apply Theorem 3.4. Since α>γ−2s, then $G\left(\delta^{\alpha }\right)≍\delta^{\gamma }$ and, therefore,

$$\begin{array}{c} \int_{\Omega }|G\left(f\right)|\;\delta^{\alpha }⩽C\int_{\Omega }\left|f\right|\;\delta^{\gamma }. \end{array}$$

This completes the proof.□

Corollary 3.10 Under the assumptions of Theorem 3.9, if γ<2s, then solutions for any admissible data are in $L^{1}\left(\Omega \right)$.

In the notations of Theorem 3.9, note that, if γ<2s, then α=0 is an admissible choice.□

For $f\in L_{c}^{\infty }\left(\Omega \right)$, we have shown that $G\left(f\right)≍\delta^{\gamma }$. To study the sharp boundary behaviour, we want to study $G\left(f\right)/\delta^{\gamma }$. For this reason, we introduce the following definition.

Definition 3.11 We denote by

$$D_{\gamma }u\left(z\right):=\lim_{\begin{array}{c} x\to z\\ x\in \Omega \end{array}}\frac{u(x)}{\delta {\left(x\right)}^{\gamma }}\;z\in \partial \Omega ,$$

and we call it γ‐normal derivative of u.

To prove sharp boundary behaviour we assume that the Green kernel has a γ‐normal derivative $D_{\gamma }G$.

(K5) $$\begin{array}{cc} & \text{There}\;\text{exists}\;D_{\gamma }G:\partial \Omega \times \Omega \to R,\;\text{such}\;\text{that,}\;\text{for}\;\text{every}\;\text{sequence}\;\Omega ∋x_{j}\to z\in \partial \Omega \\ & \text{we}\;\text{have}\;\lim_{j}\frac{G(x_{j},\cdot )}{\delta {\left(x_{j}\right)}^{\gamma }}=D_{\gamma }G\left(z,\cdot \right)\;\text{a.e.}\;\text{in}\;\Omega . \end{array}$$

Remark 3.12 Note that $D_{\gamma }G\left(z,y\right)=D_{\gamma }\left(G\left(\cdot ,y\right)\right)\left(z\right)$.

Remark 3.13 As a consequence of (K2) we have, for a.e. y∈Ω and z∈∂Ω,

(3.8) $$\begin{array}{cc} D_{\gamma }G\left(z,y\right) & ≍\lim_{\begin{array}{c} x\to z\\ x\in \Omega \end{array}}\frac{1}{{\left|x-y\right|}^{n-2s}}{\left(\frac{\delta (y)}{{\left|x-y\right|}^{2}}\wedge \frac{1}{\delta (x)}\right)}^{\gamma }=\frac{{\delta (y)}^{\gamma }}{{\left|z-y\right|}^{n-2s+2\gamma }}. \end{array}$$

Remark 3.14 Assumption (K5) is satisfied in our three reference examples.

- For the RFL, it follows from the Boundary Harnack Principle [5] and the boundary regularity of solutions on smooth domains [37]; for $y\in \Omega ,\psi \in C_{c}^{\infty }\left(\Omega \right)$ fixed and γ=s, we have
$$\begin{array}{c} \frac{G(x,y)}{\delta {\left(x\right)}^{s}}=\frac{G(x,y)}{G(\psi )(x)}\frac{G(\psi )(x)}{\delta {\left(x\right)}^{s}},\;x\in \Omega . \end{array}$$
  Both factors lie in $C^{\alpha }\left(\bar{\Omega }\setminus B_{r}\left(y\right)\right)$, at least for α,r>0 small enough. Indeed, the first one is due to [5, Theorem 1] and is a consequence of the s‐harmonicity of the two involved functions close to the boundary; the second factor, instead, is more related to the smoothness of the boundary and a more classical Schauder regularity, see [37, Theorem 1.2]. The kernel $D_{\gamma }G$ has been first introduced in [1], although it is strongly related to the Martin kernel, see, for example, [6].
- For the SFL, the well‐definition of $D_{\gamma }G$ is contained in [2, Lemma 14]; in this case, the proof relies on a computation on an explicit representation formula for G in terms of the classical Dirichlet heat kernel.
- For the CFL, a Boundary Harnack Inequality is available (see [7, section 6]), so we can repeat the argument for the RFL. To this regard see, in particular, [7, Remark 6.1 and equation (6.35)].

Theorem 3.15 Assume (K0)–(K3) and (K5) and let $f\in L_{c}^{1}\left(\Omega \right)$. For u=G(f), $D_{\gamma }u$ is well‐defined on ∂Ω. Furthermore,

$$D_{\gamma }u\left(z\right)=\int_{\Omega }D_{\gamma }G\left(z,y\right)\;f\left(y\right)\;dy,\;z\in \partial \Omega ,$$

and

$$|D_{\gamma }{u\left(z\right)|⩽C∥f∥}_{L^{1}\left(\Omega \right)}\;\mathrm{dist}{\left(z,\mathrm{supp}(f)\right)}^{2s-2\gamma -n},\;z\in \partial \Omega .$$

We write

$$\frac{u(x)}{{\delta (x)}^{\gamma }}=\int_{\Omega }\frac{G(x,y)}{{\delta (x)}^{\gamma }}\;f\left(y\right)\;dy,\;x\in \Omega .$$

Let z∈∂Ω, ${\left(x_{j}\right)}_{j\in N}\subset \Omega$ such that $x_{j}\to z$ as j↑∞, and K=supp(f)⋐Ω. Then, up to constants, for j sufficiently large

$$\frac{G(x_{j},y)}{{\delta (x_{j})}^{\gamma }}⩽\text{dist}{\left(x_{j},K\right)}^{2s-n-2\gamma }\delta {\left(y\right)}^{\gamma }⩽\mathrm{dist}{\left(K,\partial \Omega \right)}^{2s-n-2\gamma }\;y\in K.$$

Therefore, since convergence a.e. in y is given by (K5), by dominated convergence

$$\frac{G(x_{j},\cdot )}{\delta {\left(x_{j}\right)}^{\gamma }}\;f\to D_{\gamma }G\left(z,\cdot \right)f\;\;\text{in}\;L^{1}\left(\Omega \right),\;\text{as}\;j↑\infty .$$

Thus,

$$\frac{u(x_{j})}{{\delta (x_{j})}^{\gamma }}=\int_{\Omega }\frac{G(x_{j},y)}{{\delta (x_{j})}^{\gamma }}f\left(y\right)\;dy\to \int_{\Omega }D_{\gamma }G\left(z,y\right)f\left(y\right)\;dy\;\text{as}\;j↑\infty .$$

The limit is, by definition, $D_{\gamma }u\left(z\right)$. The pointwise estimate is a consequence of (3.8).□

Remark 3.16 One needs to be careful with pathological cases that do not satisfy (K5), including

$$G\left(x,y\right)=\left(2+\sin\frac{1}{\delta (x)}\right)\left(2+\sin\frac{1}{\delta (y)}\right)\frac{1}{{\left|x-y\right|}^{n-2s}}{\left(\frac{\delta (x)\delta (y)}{{\left|x-y\right|}^{2}}\wedge 1\right)}^{\gamma }.$$

Remark 3.17 Due to the lower Hopf estimates in Theorem 2.6, if f⩾0 we have

$$D_{\gamma }u\left(z\right)⩾c\int_{\Omega }f\left(y\right)\;\delta {\left(y\right)}^{\gamma }\;dy,\;z\in \partial \Omega .$$

Proposition 3.18 Assume (K0)–(K3) and (K5). Let j∈N, $A_{j}=\left\{1/j<\delta <2/j\right\}$, u=G(f) for some $f\in L_{c}^{\infty }\left(\Omega \right)$, and h be continuous on a neighbourhood of ∂Ω. Then

$$\frac{1}{|A_{j}|}\int_{A_{j}}h\left(x\right)\;\frac{u(x)}{\delta {\left(x\right)}^{\gamma }}\;dx⟶\frac{1}{\left|\partial \Omega \right|}\int_{\partial \Omega }h\left(z\right)\;D_{\gamma }u\left(z\right)\;dz,\;\text{as}\;j↑\infty .$$

We write

$$\begin{array}{c} \frac{1}{|A_{j}|}\int_{A_{j}}h\left(x\right)\frac{u(x)}{\delta {\left(x\right)}^{\gamma }}\;dx=\frac{j}{|A_{j}|}\int_{\partial \Omega }\frac{1}{j}\int_{1/j}^{2/j}h\left(z+\rho n\left(z\right)\right)\frac{u(z+\rho n(z))}{\rho^{\gamma }}\;d\rho \;dz. \end{array}$$

Therefore, for every z∈∂Ω,

$$I_{j}\left(z\right)=\frac{1}{j}\int_{1/j}^{2/j}h\left(z+\rho n\left(z\right)\right)\frac{u(z+\rho n(z))}{\rho^{\gamma }}d\rho \;⟶\;h\left(z\right)D_{\gamma }u\left(z\right),\;\text{as}\;j↑\infty .$$

Since $I_{j}$ is a bounded function in ∂Ω and pointwise convergent, by the dominated convergence theorem as j↑∞

$$\frac{j}{|A_{j}|}\int_{\partial \Omega }\frac{1}{j}\int_{1/j}^{2/j}h\left(z+\rho n\left(z\right)\right)\frac{u(z+\rho n(z))}{\rho^{\gamma }}\;d\rho \;dz\;⟶\;\frac{1}{\left|\partial \Omega \right|}\int_{\partial \Omega }h\left(z\right)D_{\gamma }u\left(z\right)\;dz.$$

This completes the proof.□

## 4 LIMIT OF THE INTERIOR THEORY: THE L‐HARMONIC PROBLEM

### 4.1 Limit of the interior theory

A classical approach known for the usual Laplacian to recover the non‐homogeneous Dirichlet boundary problem is to concentrate all mass towards the boundary.

Let us recall a classical argument in the case of the usual Laplacian on the ball of radius 1. To construct a harmonic function such that u=1 on the boundary of the ball we can proceed as follows. Taking a well‐chosen sequence $f_{j}$ of radial functions such that $∥f_{j}{\delta ∥}_{L^{1}\left(\Omega \right)}\to \left|\partial \Omega \right|$ but such that support of $f_{j}$ is contained in the strip $\left\{1-\frac{1}{j}<r<1\right\}$, one can pass to the limit $G(f_{j})$ by compactness. Looking at the very‐weak formulation (or weak‐dual one) we can check that the limit is the desired function. We now extend this argument to our general setting, and general Ω.

Theorem 4.1 Let G satisfy (K0)–(K3) and (K5). Let $A_{j}=\left\{1/j<\delta <2/j\right\}$, j∈N, and

$$\begin{array}{c} f_{j}=\frac{|\partial \Omega |\;\chi_{A_{j}}}{|A_{j}|\;\delta^{\gamma }} \end{array}$$

such that $∥f_{j}\delta^{\gamma }{∥}_{L^{1}}=\left|\partial \Omega \right|$. Then, there exists a function in $u^{★}\in L_{\text{loc}}^{1}\left(\Omega \right)$ such that

$$G\left(f_{j}\right)⇀u^{★},\;\;\text{in}\;L^{1}\left(K\right)\mathrm{forevery}K⋐\Omega .$$

Furthermore, $u^{★}$ is a solution of

$$\int_{\Omega }u^{★}\psi =\int_{\partial \Omega }D_{\gamma }\left(G(\psi )\right),\;\text{for}\;\text{any}\;\psi \in L_{c}^{\infty }\left(\Omega \right)$$

and is given by

(4.1) $$u^{★}\left(x\right)=\int_{\partial \Omega }D_{\gamma }G\left(z,x\right)\;dz,\;x\in \Omega .$$

It is clear that $\mathrm{supp}\left(f_{j}\right)={\bar{A}}_{j}$ and $∥f_{j}\delta^{\gamma }{∥}_{L^{1}\left(\Omega \right)}=\left|\partial \Omega \right|$. Therefore, due to Lemma 2.9, a subsequence of $G(f_{j})$, $G(f_{j}^{\left(1\right)})$, is weakly convergent in $L^{1}\left(\left\{\delta ⩾1\right\}\right)$ to a function $u_{1}$. A further subsequence, $G(f_{j}^{\left(2\right)})$, converges in $L^{1}\left(\left\{\delta ⩾\frac{1}{2}\right\}\right)$ to a function $u_{2}$. Iterating the process, we construct sequences $f_{j}^{\left(m\right)}$ and functions $u_{m}$ defined on {δ>1/m}, for every m∈N. Applying Proposition 3.18 we have that

$$\begin{array}{cc} \int_{\Omega }G\left(f_{j}\right)\psi & =\int_{\Omega }G\left(\psi \right)\frac{|\partial \Omega |\;\chi_{A_{j}}}{|A_{j}|\;\delta^{\gamma }}=\frac{\left|\partial \Omega \right|}{|A_{j}|}\int_{A_{j}}\frac{G(\psi )}{\delta^{\gamma }}\to \int_{\partial \Omega }D_{\gamma }\left[G\left(\psi \right)\right] \end{array}$$

for any $\psi \in L_{c}^{\infty }\left(\Omega \right)$. Therefore,

$$\int_{\Omega }u_{m}\psi =\int_{\partial \Omega }D_{\gamma }\left[G\left(\psi \right)\right],\;\text{for}\;\text{any}\;\psi \in L_{c}^{\infty }\left(\Omega \right)\;\text{such}\;\text{that}\;\mathrm{supp}\psi \subseteq \left\{\delta ⩾1/m\right\}.$$

For m>k, using $\psi =\mathrm{sign}\left(u_{m}-u_{k}\right)\chi_{\left\{\delta ⩾1/k\right\}}$ as a test function, we check that $u_{m}{|}_{\delta ⩾1/k}=u_{k}$. We define $u^{★}\left(x\right)=u_{m}\left(x\right)$ for any m>1/δ(x). Given $\psi \in L_{c}^{\infty }\left(\Omega \right)$, we $u^{★}\psi =u_{m}\psi \;$ for any m>1/dist(suppψ,∂Ω). Therefore,

$$\begin{array}{cc} \int_{\Omega }u^{★}\psi & =\int_{\partial \Omega }D_{\gamma }\left[G\left(\psi \right)\right]\;\;\text{for}\;\text{any}\;\psi \in L_{c}^{\infty }\left(\Omega \right). \end{array}$$

If we now consider the Green representation, we get

$$\int_{\partial \Omega }D_{\gamma }\left[G\left(\psi \right)\right]\;dz=\int_{\partial \Omega }\left(\int_{\Omega }D_{\gamma }G\left(z,x\right)\;\psi \left(x\right)\;dx\right)dz=\int_{\Omega }\psi \left(x\right)\left(\int_{\partial \Omega }D_{\gamma }G\left(z,x\right)\;dz\right)dx.$$

With this representation formula, we show that all convergent subsequences share a limit, and therefore the whole sequence converges.□

**FIGURE 4:**
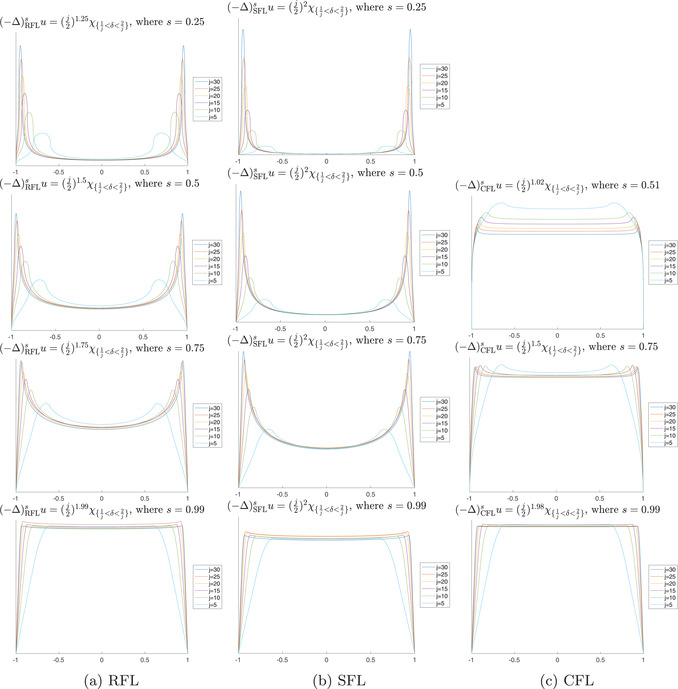
Numerical solutions of $Lu={\left(\frac{j}{2}\right)}^{1+\gamma }\chi_{\frac{1}{j}<\delta <\frac{2}{j}}$ in dimension n=1. In the limit as j↑+∞, we recover the profile of solutions to the L‐harmonic problem. We implement the schemes introduced in Figure 3.

Remark 4.2 In Figure 4, we show a numerical simulation of the behaviour of the approximating sequence for the case of the SFL, under different values of s.

Corollary 4.3 Under the assumptions and notations of Theorem 4.1, it holds

(4.2) $$u^{★}≍\left\{\begin{array}{cc} \delta^{2s-\gamma -1} & \gamma >s-\frac{1}{2}\\ \delta^{\gamma }\;\left(1+|\;\ln\delta |\right) & \gamma =s-\frac{1}{2}\\ \delta^{\gamma } & \gamma <s-\frac{1}{2} \end{array}\right.\;\text{in}\;\Omega .$$

This follows by plugging (3.8) into (4.1). Indeed,

$$\begin{array}{c} u^{★}\left(x\right)≍{\delta (x)}^{\gamma }\int_{\partial \Omega }\frac{dz}{{\left|z-x\right|}^{n-2s+2\gamma }},\;x\in \Omega , \end{array}$$

where

$$\begin{array}{c} \int_{\partial \Omega }\frac{dz}{{\left|z-x\right|}^{n-2s+2\gamma }}≍\left\{\begin{array}{cc} \delta {\left(x\right)}^{2s-2\gamma -1} & 2s-2\gamma -1<0\\ 1+|\;\ln\delta (x)| & 2s-2\gamma -1=0\\ 1 & 2s-2\gamma -1>0 \end{array}\right.\;x\in \Omega , \end{array}$$

which completes the proof.□

Remark 4.4 Note that, for $\gamma >s-\frac{1}{2}$, $u^{★}$ has the limit rate $\delta^{2s-\gamma -1}$ which is not accessible to solutions of the interior problem.

Remark 4.5 The function $u^{★}$ is a large solution (that is, $u^{★}\left(x\right)↑+\infty$ as δ(x)↓0) if and only if 2s−γ−1<0. We have:

(1) in the RFL case γ=s, so 2s−γ−1=s−1<0;
(2) in the SFL case γ=1, so 2s−γ−1=2(s−1)<0;
(3) in the CFL case $\gamma =s-\frac{1}{2}$, so 2s−γ−1=0 for $\frac{1}{2}<s<1$; in this case $u^{★}$ is not singular.

### 4.2 The L‐harmonic problem

For a self‐adjoint operator in our class of study it makes sense to consider the following boundary problem

(4.3) $$\int_{\Omega }u\;L\varphi =\int_{\partial \Omega }h\;D_{\gamma }\varphi$$

for some suitable test functions φ. In the case of the usual Laplacian, this is the non‐homogeneous Dirichlet problem with data h. This very weak formulation was first studied in [12].

Passing to our weak‐dual formulation, (4.3) is written

(4.4) $$\int_{\Omega }u\psi =\int_{\partial \Omega }h\;D_{\gamma }\left[G\left(\psi \right)\right]\;\text{for}\;\text{any}\;\psi \in L_{c}^{\infty }\left(\Omega \right).$$

Heuristically, (4.4) can be read as the L‐harmonicity of u in Ω, that is, Lu=0 in Ω.

To understand this weak‐dual problem, we proceed informally. If one takes $\psi =\mu_{x}$, the Dirac delta, we obtain

$$u\left(x\right)=\int_{\partial \Omega }h\left(z\right)\;D_{\gamma }G\left(z,x\right)\;dz.$$

We will see in Theorem 4.13 that

$$\frac{u(x)}{u^{★}\left(x\right)}=\int_{\partial \Omega }h\left(z\right)\frac{D_{\gamma }G\left(z,x\right)}{u^{★}\left(x\right)}\;dz⟶h\left(\theta \right).$$

Hence, in some sense (4.4) is a formulation of problem (1.8). We will devote the next paragraphs of this section to rigorously proving these intuitions.

### 4.3 Existence, uniqueness, and kernel representation

We have the following theorem of well‐posedness.

Theorem 4.6 Let Ω be a smooth domain, assume (K0)–(K3) and (K5) and $h\in L^{1}\left(\partial \Omega \right)$. Then, there exists a unique $u\in L_{\text{loc}}^{1}\left(\Omega \right)$ satisfying (4.4). Furthermore,

(1) this unique solution can be represented by
(4.5) $$u\left(x\right)=\int_{\partial \Omega }D_{\gamma }G\left(z,x\right)\;h\left(z\right)\;dz,\;\text{for}\;x\in \Omega ;$$
(2) we have the estimate
(4.6) $$\begin{array}{cc} {∥u∥}_{L^{\infty }\left(K\right)} & ⩽C\;\mathrm{dist}{\left(K,\partial \Omega \right)}^{2s-n-\gamma }{∥h∥}_{L^{1}\left(\partial \Omega \right)}; \end{array}$$
(3) if h∈C(∂Ω), then there exists a sequence ${\left(f_{j}\right)}_{j\in N}\subset L^{1}\left(\Omega ,\delta^{\gamma }\right)$ such that
(4.7) $$G\left(f_{j}\right)⇀u\;\;\text{in}\;L_{\text{loc}}^{1}\left(\Omega \right),\;\text{as}\;j↑\infty .$$

The uniqueness is immediate to prove. Let $u_{1},u_{2}$ be two solutions, then $u=u_{1}-u_{2}$ satisfies

$$\int_{\Omega }u\psi =0\;\text{for}\;\text{any}\;\psi \in L_{c}^{\infty }\left(\Omega \right).$$

In particular, let K⋐Ω and take $\psi =\mathrm{sign}\left(u\right)\chi_{K}$. Then

$$\int_{K}\left|u\right|=0.$$

Since this is true for all K, we have that u=0 a.e. in Ω. Hence $u_{1}=u_{2}$.

The kernel representation (4.5) follows as in the proof of Theorem 4.1, by exchanging the order of integration in (4.4). Note that this kernel representation can be rigorously justified on its own and therefore grant uniqueness. Nevertheless, since we will construct it as a limit of the interior theory, this is not needed.

We prove existence, (4.6), and (4.7) simultaneously. We split the proof into different steps.

Let us first assume that 0⩽h∈C(∂Ω). Using the notations defined in Remark 3.1, we extend the definition of h to the interior by setting

H(x)=h(z(x)).

Recall that δ(x)=|x−z(x)|,z(x)∈∂Ω, and note that H∈C({δ<ε}).

Let us define, for $j>\frac{1}{\varepsilon }$, the sequence

$$f_{j}=H\;\frac{\left|\partial \Omega \right|\chi_{A_{j}}}{|A_{j}|\delta^{\gamma }}\;\in L^{1}\left(\Omega \right).$$

We check that this sequence is bounded in $L^{1}\left(\Omega ,\delta^{\gamma }\right)\;$ by estimating

$$\int_{\Omega }f_{j}\delta^{\gamma }=\int_{A_{j}}\frac{\left|\partial \Omega \right|}{|A_{j}|}H=\frac{\left|\partial \Omega \right|}{|A_{j}|}\int_{A_{j}}H⩽{|\partial \Omega |∥H∥}_{L^{\infty }\left(\Omega \right)}.$$

We define $u_{j}=G\left(f_{j}\right)$.

We now show local $L^{1}$‐weak convergence. Let K⋐Ω. For any A⊂K we have that

$$\int_{A}u_{j}⩽C_{K,\beta }{\left|A\right|}^{\beta }∥f_{j}\delta^{\gamma }{∥}_{L^{1}\left(\Omega \right)}⩽C_{K,\beta }{∥H∥}_{L^{\infty }\left(\Omega \right)}{\left|A\right|}^{\beta },$$

for some β>0, by Lemma 2.9. Therefore, the sequence $u_{j}$ is equi‐integrable in K and it admits a subsequence $u_{j_{k}}$ weakly convergent to some $u_{K}\in L^{1}\left(K\right)$. That is, if we consider $\psi \in L^{\infty }\left(\Omega \right)$, with suppψ⊆K, we have that

$$\int_{\Omega }u_{j_{k}}\psi \to \int_{\Omega }u_{K}\psi ,\;\text{as}\;k↑\infty .$$

On the other hand, by Proposition 3.18, we have that

$$\begin{array}{cc} \int_{\Omega }G\left(\psi \right)f_{j} & =\frac{\left|\partial \Omega \right|}{|A_{j}|}\int_{A_{j}}\frac{G(\psi )}{\delta^{\gamma }}\;H⟶\int_{\partial \Omega }D_{\gamma }\left[G\left(\psi \right)\right]\;h. \end{array}$$

Therefore,

$$\int_{\Omega }u_{K}\psi =\int_{\partial \Omega }D_{\gamma }\left[G\left(\psi \right)\right]h,\;\text{for}\;\text{any}\;\psi \in L^{\infty },\;\text{with}\;\mathrm{supp}\psi \subseteq K.$$

For two compacts $K,K^{\prime }⋐\Omega$ and the corresponding $u_{K},u_{K^{\prime }}$ built as above, we actually have $u_{K}=u_{K^{\prime }}$ in $K\cap K^{\prime }$. Indeed, let us consider the test function

$$\psi =\left\{\begin{array}{cc} \mathrm{sign}(u_{K}-u_{K^{\prime }}) & \text{in}\;K\cap K^{\prime },\\ 0 & \text{in}\;\Omega \setminus (K\cap K^{\prime }). \end{array}\right.$$

It is an admissible test function for both $u_{K}$ and $u_{K^{\prime }}$. Therefore,

$$\int_{K\cap K^{\prime }}\left|u_{K}-u_{K^{\prime }}\right|=0.$$

We define now

$$u\left(x\right)=u_{K(x)}\left(x\right)\;x\in \Omega ,\;\text{where}\;K\left(x\right)=\left\{y\in \Omega :\delta \left(y\right)⩾\delta \left(x\right)/2\right\}.$$

We have shown above that any converging subsequence of $u_{j}$ converges weakly to u over compacts. In particular, $u_{j}⇀u$ in $L_{\text{loc}}^{1}$. By construction u solves (4.4).

Passing to the limit the estimate in Theorem 2.5

$$\int_{K}u_{j}⩽C_{K}{∥f_{j}\delta^{\gamma }∥}_{L^{1}\left(\Omega \right)},$$

we deduce that, as j↑∞,

$$\int_{K}u⩽C_{K}{∥h∥}_{L^{1}\left(\partial \Omega \right)}.$$

Moreover, in view of (2.5) we have that

$$∥u_{j}{∥}_{L^{\infty }\left(K\right)}⩽C\mathrm{dist}{\left(K,A_{j}\right)}^{2s-n-\gamma }{∥f_{j}\delta^{\gamma }∥}_{L^{1}\left(\Omega \right)}.$$

We deduce that the sequence $u_{j}$ converges weak‐★ in $L^{\infty }\left(K\right)$ to u, and that

$${∥u∥}_{L^{\infty }\left(K\right)}⩽C\mathrm{dist}{\left(K,\partial \Omega \right)}^{2s-n-\gamma }{∥h∥}_{L^{1}\left(\partial \Omega \right)}.$$

We now consider $0⩽h\in L^{1}\left(\partial \Omega \right)$. We take an approximation sequence $0⩽h_{k}\in C\left(\partial \Omega \right)$ converging to h in $L^{1}\left(\partial \Omega \right)$. The sequence $u_{k}$ of solutions corresponding to $h_{k}$ can be constructed through the previous step. Due to the estimates, we can pass to the limit over compacts and apply the uniqueness reasoning above to recover a function $u\in L_{\text{loc}}^{\infty }$ solution of (4.4) with data h.

For $h\in L^{1}\left(\partial \Omega \right)$, we can decompose it as $h=h_{+}-h_{-}$, construct solutions $u_{1}$ and $u_{2}$ corresponding to $h_{+}$ and $h_{-}$ and recover $u=u_{1}-u_{2}$ satisfying all properties.

This completes the proof.□

Corollary 4.7 In the assumptions of Theorem 4.6 and for u defined as in (4.5), we have that

$${\left\|\frac{u}{u^{★}}\right\|}_{L^{1}\left(\Omega \right)}⩽C{∥h∥}_{L^{1}\left(\partial \Omega \right)}.$$

It holds

$$\begin{array}{c} \int_{\Omega }\frac{\left|u(x)\right|}{u^{★}\left(x\right)}\;dx⩽\int_{\partial \Omega }\left|h\left(z\right)\right|\int_{\Omega }\frac{D_{\gamma }G\left(z,x\right)}{u^{★}\left(x\right)}\;dx\;dz. \end{array}$$

In view of (3.8) and (4.2), for z∈∂Ω,

$$\begin{array}{c} \int_{\Omega }\frac{D_{\gamma }G\left(z,x\right)}{u^{★}\left(x\right)}\;dx≍\left\{\begin{array}{cccc} & \int_{\Omega }\frac{\delta {\left(x\right)}^{2\gamma -2s+1}}{{\left|x-z\right|}^{n-2s+2\gamma }}\;dx & & \text{if}\;\gamma >s-\frac{1}{2},\\ & \int_{\Omega }\frac{dx}{{\left|x-z\right|}^{n-1}\;\left(1+|\;\ln\delta (x)|\right)} & & \text{if}\;\gamma =s-\frac{1}{2},\\ & \int_{\Omega }\frac{dx}{{\left|x-z\right|}^{n-2s+2\gamma }} & & \text{if}\;\gamma <s-\frac{1}{2}. \end{array}\right. \end{array}$$

When γ<s−1/2, then 2γ−2s<−1, which implies

$$\begin{array}{c} \int_{\Omega }\frac{dx}{{\left|x-z\right|}^{n-2s+2\gamma }}≍1,\;\text{on}\;\partial \Omega . \end{array}$$

When γ⩾s−1/2, it suffices to use relation δ(x)⩽|x−z| for any x∈Ω,z∈∂Ω in order to deduce

$$\begin{array}{c} \int_{\Omega }\frac{\delta {\left(x\right)}^{2\gamma -2s+1}}{{\left|x-z\right|}^{n-2s+2\gamma }}\;dx⩽\int_{\Omega }\frac{dx}{{\left|x-z\right|}^{n-1}}≍1,\;\text{on}\;\partial \Omega , \end{array}$$

and

$$\int_{\Omega }\frac{dx}{{\left|x-z\right|}^{n-1}\;\left(1+|\;\ln\delta (x)|\right)}⩽\int_{\Omega }\frac{dx}{{\left|x-z\right|}^{n-1}\;\left(1+|\;\ln|x-z||\right)}≍1,\;\text{on}\;\partial \Omega .$$

□

Corollary 4.8 Under the assumptions of Theorem 4.6, the solution operator to problem (4.4)

(4.8) $$M:L^{1}\left(\partial \Omega \right)\to L_{\text{loc}}^{\infty }\left(\Omega \right)$$

is linear, continuous, and it admits the kernel representation

(4.9) $$M\left(h\right)\left(x\right)=\int_{\partial \Omega }M\left(x,z\right)\;h\left(z\right)\;dz,\;x\in \Omega ,$$

where M is given by

(4.10) $$M\left(x,z\right)=D_{\gamma }G\left(z,x\right).$$

Furthermore, for any α>(γ−2s)∨(−γ−1)

(4.11) $$M:L^{1}\left(\partial \Omega \right)\to L^{1}\left(\Omega ,\delta^{\alpha }\right)\;\text{is}\;\text{continuous}$$

with operator norm

$${∥M∥}_{L^{1}\left(\partial \Omega \right);L^{1}\left(\Omega ,\delta^{\alpha }\right)}⩽{∥G∥}_{L^{1}\left(\Omega ,\delta^{\gamma }\right);L^{1}\left(\Omega ,\delta^{\alpha }\right)}.$$

The results (4.8), (4.9), and (4.10) follow immediately from Theorem 4.6.

Now, let us prove (4.11). First, let 0⩽h∈C(∂Ω). By recalling the construction in Theorem 4.6, there is a sequence $f_{j}⩾0$ such that $0⩽u_{j}=G\left(f_{j}\right)$ with $∥f_{j}\delta^{\gamma }{∥}_{L^{1}\left(\Omega \right)}={∥h∥}_{L^{1}\left(\partial \Omega \right)}$ such that $u_{j}⇀u$ in $L_{\text{loc}}^{1}\left(\Omega \right)$. Going back to the proof of Theorem 3.9 we observe that

$$\int_{\Omega }u_{j}\delta^{\alpha }⩽C_{G}\int_{\Omega }f_{j}\;\delta^{\gamma }=C_{G}\int_{\partial \Omega }h,$$

where $C_{G}:={∥G∥}_{L^{1}\left(\Omega ,\delta^{\gamma }\right);L^{1}\left(\Omega ,\delta^{\alpha }\right)}$. Since we only have convergence over compact sets, we assure that, for any K⋐Ω,

$$\int_{K}u_{j}\delta^{\alpha }⩽C_{G}\int_{\partial \Omega }h.$$

Since $\chi_{K}\delta^{\alpha }\in L_{c}^{\infty }\left(\Omega \right)$ and $u_{j}$ converges weakly in $L_{\text{loc}}^{1}\left(\Omega \right)$

$$\int_{K}u\delta^{\alpha }=\lim_{j↑+\infty }\int_{K}u_{j}\delta^{\alpha }⩽C_{G}\int_{\partial \Omega }h.$$

Since this holds for any K⋐Ω and $C_{G}$ does not depend on K, then $u\delta^{\alpha }\in L^{1}\left(\Omega \right)$ and

(4.12) $$\int_{\Omega }u\delta^{\alpha }⩽C_{G}\int_{\partial \Omega }h.$$

If $0⩽h\in L^{1}\left(\partial \Omega \right)$, we can construct an approximating sequence $0⩽h_{j}\in C\left(\partial \Omega \right)$ and we recover (4.12) by passing to the limit.

If $h\in L^{1}\left(\partial \Omega \right)$ is sign‐changing, we repeat the argument for $h_{+}$ and $h_{-}$ and apply (4.12) to deduce

$$\int_{\Omega }\left|u\right|\delta^{\alpha }⩽C_{G}\int_{\partial \Omega }\left|h\right|.$$

This completes the proof.□

Remark 4.9 Let u=M(h). Note that, since 2s−γ−1<0, (4.11) shows that $u\delta^{\varepsilon +\gamma -2s}\in L^{1}\left(\Omega \right)$ for any ε>0. This is sharper than Corollary 4.7, which only guarantees that $u\delta^{1+\gamma -2s}≍u/u^{*}\in L^{1}\left(\Omega \right)$.

Remark 4.10 Due to the estimates for $D_{\gamma }G$, we know that

$$M\left(x,z\right)≍\frac{\delta {\left(x\right)}^{\gamma }}{{\left|x-z\right|}^{n+\gamma -(2s-\gamma )}}\;x\in \Omega ,z\in \partial \Omega .$$

Remark 4.11 In the classical case, this corresponds to the usual Poisson kernel. For $L={\left(-\Delta \right)}_{\mathrm{RFL}}^{s}$, this somehow corresponds to the existing notion of Martin kernel (see [1, 6]).

Remark 4.12 Note that

$$\left|\frac{M(x,z)}{\int_{\partial \Omega }M\left(x,z^{\prime }\right)dz^{\prime }}\right|⩽C\frac{\delta {\left(x\right)}^{2\gamma +1-2s}}{{\left|x-z\right|}^{n-2s+2\gamma }}\;x\in \Omega ,z\in \partial \Omega .$$

### 4.4 Boundary behaviour of solutions of the L‐harmonic problem

#### 4.4.1 Bounded data

Theorem 4.13 Let us assume (K0)–(K3) and (K5). Let h∈C(∂Ω) and $\gamma >s-\frac{1}{2}$. Then, the unique solution $u\in L_{\text{loc}}^{1}\left(\Omega \right)$ of (4.4) satisfies

$$\begin{array}{c} \lim_{\begin{array}{c} x\to \theta \\ x\in \Omega \end{array}}\frac{u(x)}{u^{★}\left(x\right)}=h\left(\theta \right) \end{array}$$

uniformly in θ∈∂Ω.

We estimate, for x∈Ω and up to multiplicative constants,

$$\begin{array}{ccc} \left|\frac{u(x)}{u^{★}\left(x\right)}-h\left(\theta \right)\right| & = & \left|\frac{\int_{\partial \Omega }M\left(x,z\right)h\left(z\right)\;dz}{\int_{\partial \Omega }M\left(x,z^{\prime }\right)\;dz^{\prime }}-\frac{\int_{\partial \Omega }M\left(x,z\right)h\left(\theta \right)\;dz}{\int_{\partial \Omega }M\left(x,z^{\prime }\right)\;dz^{\prime }}\right|\\ & = & \left|\int_{\partial \Omega }\frac{M(x,z)}{\int_{\partial \Omega }M\left(x,z^{\prime }\right)dz^{\prime }}\left(h\left(z\right)-h\left(\theta \right)\right)\;dz\right|⩽\delta {\left(x\right)}^{-2s+2\gamma +1}\int_{\partial \Omega }\frac{\left|h(z)-h(\theta )\right|}{{\left|x-z\right|}^{n+2\gamma -2s}}\;dz, \end{array}$$

where we have used Lemma 4.10. Fix now ε>0 arbitrarily small and let η>0 small enough in order to have |h(z)−h(θ)|<ε for any z∈∂Ω∩B(θ,η). Note that, since ∂Ω is compact and h is continuous, then η is independent of θ by uniform continuity. Then we have

$$\delta {\left(x\right)}^{-2s+2\gamma +1}\int_{\partial \Omega \cap B(\theta ,\eta )}\frac{\left|h(z)-h(\theta )\right|}{{\left|x-z\right|}^{n+2\gamma -2s}}\;dz⩽\delta {\left(x\right)}^{-2s+2\gamma +1}\int_{\partial \Omega }\frac{\varepsilon }{{\left|x-z\right|}^{n+2\gamma -2s}}\;dz⩽\varepsilon$$

and

$$\delta {\left(x\right)}^{-2s+2\gamma +1}\int_{\partial \Omega \setminus B(\theta ,\eta )}\frac{\left|h(z)-h(\theta )\right|}{{\left|x-z\right|}^{n+2\gamma -2s}}\;dz⩽\varepsilon^{-n-2\gamma +2s}{∥h∥}_{L^{\infty }\left(\partial \Omega \right)}\delta {\left(x\right)}^{-2s+2\gamma +1}\;⟶\;0$$

as x→θ. The above yields that

$$\begin{array}{c} \underset{x\to \theta }{lim sup}\left|\frac{u(x)}{u^{★}\left(x\right)}-h\left(\theta \right)\right|⩽\varepsilon , \end{array}$$

but, since ε is arbitrary, our claim is proved.□

Remark 4.14 The reasoning above also holds for $\gamma =s-\frac{1}{2}$ under suitable modifications. For $\gamma <s-\frac{1}{2}$ the integration

$$\int_{\partial \Omega }{\left|\theta -z\right|}^{-n-2\gamma +2s}\;h\left(z\right)\;dz$$

makes perfect sense for θ∈∂Ω, so no normalisation will ever be able to improve

$$\lim_{x\to \theta }\delta {\left(x\right)}^{-\gamma }u\left(x\right)≍\int_{\partial \Omega }{\left|\theta -z\right|}^{-n-2\gamma +2s}\;h\left(z\right)\;dz.$$

Heuristically, it seems like that the Martin kernel is *not* singular enough to select only the values of h around θ when passing to the limit. The kernel seems to be too ‘spread around’.

#### 4.4.2 Integrable data

Theorem 4.15 Let $h\in L^{1}\left(\partial \Omega \right)$ and $\gamma ⩾s-\frac{1}{2}$. Then, for any $\phi \in C(\bar{\Omega })$, it holds

$$\frac{1}{\eta }\int_{\left\{\delta <\eta \right\}}\frac{M(h)}{u^{★}}\;\phi \;⟶\;\int_{\partial \Omega }h\;\phi \;\text{as}\;\eta ↓0.$$

Note that the claim holds for h∈C(∂Ω) by Theorem 4.13. For a general $h\in L^{1}\left(\partial \Omega \right)$, let us consider a sequence ${\left(h_{k}\right)}_{k\in N}\subset C\left(\partial \Omega \right)$ such that $∥h_{k}-h{∥}_{L^{1}\left(\partial \Omega \right)}↓0$ as k↑∞. Then split

$$\begin{array}{ccc} \left|\eta^{-1}\int_{\left\{\delta <\eta \right\}}\frac{M(h)}{u^{★}}\;\phi -\int_{\partial \Omega }h\;\phi \right| & ⩽ & \left|\eta^{-1}\int_{\left\{\delta <\eta \right\}}\frac{M\left(h\right)-M\left(h_{k}\right)}{u^{★}}\;\phi \right|\\ & & +\;\left|\eta^{-1}\int_{\left\{\delta <\eta \right\}}\frac{M(h_{k})}{u^{★}}\;\phi -\int_{\partial \Omega }h_{k}\;\phi \right|+\left|\int_{\partial \Omega }\left(h_{k}-h\right)\;\phi \right|. \end{array}$$

Fix ε>0 arbitrarily small and let k∈N large enough to have $∥h_{k}{-h∥}_{L^{1}\left(\partial \Omega \right)}<\varepsilon$. The above inequality and Theorem 4.13 entail

$$\begin{array}{c} \underset{\eta ↓0}{lim sup}\left|\eta^{-1}\int_{\left\{\delta <\eta \right\}}\frac{M(h)}{u^{★}}\;\phi -\int_{\partial \Omega }h\;\phi \right|⩽\underset{\eta ↓0}{lim sup}\left|\eta^{-1}\int_{\left\{\delta <\eta \right\}}\frac{M\left(h\right)-M\left(h_{k}\right)}{u^{★}}\;\phi \right|+\varepsilon , \end{array}$$

for any k∈N large enough. Write

$$\begin{array}{ccc} & & \eta^{-1}\int_{\left\{\delta <\eta \right\}}\frac{M\left(h\right)-M\left(h_{k}\right)}{u^{★}}\;\phi \\ & & \;=\eta^{-1}\int_{\left\{\delta <\eta \right\}}\frac{\phi (x)}{u^{★}\left(x\right)}\int_{\partial \Omega }M\left(x,z\right)\;\left(h_{k}\left(z\right)-h\left(z\right)\right)\;dz\;dx\\ & & \;=\int_{\partial \Omega }\left(h_{k}\left(z\right)-h\left(z\right)\right)\eta^{-1}\int_{\left\{\delta <\eta \right\}}M\left(x,z\right)\;\frac{\phi (x)}{u^{★}\left(x\right)}\;dx\;dz, \end{array}$$

in order to deduce that, up to constants, it holds

$$\begin{array}{cc} & \left|\eta^{-1}\int_{\left\{\delta <\eta \right\}}\frac{M\left(h\right)-M\left(h_{k}\right)}{u^{★}}\;\phi \right|\\ & \;⩽{∥\phi ∥}_{L^{\infty }\left(\Omega \right)}{∥h_{k}-h∥}_{L^{1}\left(\partial \Omega \right)}\underset{z\in \partial \Omega }{\sup}\eta^{-1}\int_{\left\{\delta <\eta \right\}}\frac{M(x,z)}{u^{★}\left(x\right)}\;dx\\ & \;⩽{∥\phi ∥}_{L^{\infty }\left(\Omega \right)}{∥h_{k}-h∥}_{L^{1}\left(\partial \Omega \right)}\underset{z\in \partial \Omega }{\sup}\eta^{-1}\int_{\left\{\delta <\eta \right\}}\frac{\delta {\left(x\right)}^{-2s+2\gamma +1}}{{\left|x-z\right|}^{n+2\gamma -2s}}\;dx. \end{array}$$

By the co‐area formula, it holds

$$\begin{array}{ccc} \int_{\left\{\delta <\eta \right\}}\frac{\delta {\left(x\right)}^{-2s+2\gamma +1}}{{\left|x-z\right|}^{n+2\gamma -2s}}\;dx & = & \int_{0}^{\eta }t^{-2s+2\gamma +1}\int_{\left\{x:\delta (x)=t\right\}}{\left|x-z\right|}^{-n-2\gamma +2s}\;dx\\ & = & \int_{0}^{\eta }t^{-2s+2\gamma +1}\;t^{-1-2\gamma +2s}\;dt=\eta \end{array}$$

and therefore

$$\begin{array}{c} \left|\eta^{-1}\int_{\left\{\delta <\eta \right\}}\frac{M\left(h\right)-M\left(h_{k}\right)}{u^{★}}\;\phi \right|⩽\varepsilon {∥\phi ∥}_{L^{\infty }\left(\Omega \right)}. \end{array}$$

The case $\gamma =s-\frac{1}{2}$ follows by a similar argument.□

## 5 COMMENTS AND OPEN PROBLEMS

(1) Let u=0 in $\Omega^{c}$ and let Lu=f and $h=\lim_{x\to \partial \Omega }u\left(x\right)/u^{★}\left(x\right)$. For $\psi \in L_{c}^{\infty }\left(\Omega \right)$, we have
$$\int_{\Omega }u\psi =\int_{\Omega }f\;G\left(\psi \right)+\int_{\partial \Omega }h\;D_{\gamma }\left[G\left(\psi \right)\right].$$
  Let φ=G[ψ]. The above formally gives the integration by parts formula
$$\int_{\Omega }u\;L\varphi =\int_{\Omega }\varphi \;Lu+\int_{\partial \Omega }\left(\lim_{x\to \partial \Omega }\frac{u(x)}{u^{★}\left(x\right)}\frac{\varphi (x)}{\delta {\left(x\right)}^{\gamma }}\right).$$
  Note that $u^{★}\left(x\right)\delta {\left(x\right)}^{\gamma }≍\delta {\left(x\right)}^{2s-1}$.
(2) An interesting question is what general classes of operators L lead to Green functions with estimates of type (K2). The examples presented in Subsection 1.2 come from Markov processes, in particular Lévy‐flight processes with different conditions outside Ω. However, as we presented above, their pointwise formulae are significantly different. More general examples may exist. It would be interesting to see if strange examples could be constructed, for example, an operator L such that G is given by the example in Remark 3.16.
(3) The case 2s−γ−1>γ (that is, $\gamma <s-\frac{1}{2}$) seems to pose problems to uniqueness. Indeed in this case
$$M\left(1\right)≍\delta^{(2s-\gamma -1)\wedge \gamma }⟶0\;\;\text{as}\;x\to \partial \Omega .$$
  It seems that problem
$$\left\{\begin{array}{cc} Lu=f & \text{in}\;\Omega \\ u=0 & \text{on}\;\partial \Omega \end{array}\right.$$
  does not have a unique solution, as G(f)+M(h) is also a solution for any h∈C(∂Ω). Therefore, the construction of the Green operator assumed at the beginning (which chooses a single solution), seems to be made by applying some additional *selection criteria*. This phenomenon should be studied.
(4) In trying to construct an example satisfying $\gamma <s-\frac{1}{2}$ relation, we have considered the following example: let $f\in L_{c}^{\infty }\left(\Omega \right)$ and consider the system
$$\left\{\begin{array}{cccc} {\left(-\Delta \right)}_{\mathrm{RFL}}^{\frac{s}{k}}v_{1} & =f & & \text{in}\;\Omega \\ {\left(-\Delta \right)}_{\mathrm{RFL}}^{\frac{s}{k}}v_{1} & =v_{2} & & \text{in}\;\Omega \\ & \;\vdots & & \\ {\left(-\Delta \right)}_{\mathrm{RFL}}^{\frac{s}{k}}v_{k-1} & =v_{k-2} & & \text{in}\;\Omega \\ {\left(-\Delta \right)}_{\mathrm{RFL}}^{\frac{s}{k}}u & =v_{k-1} & & \text{in}\;\Omega \\ v_{1}=\cdots =v_{k-1}=u & =0 & & \text{in}\;R^{n}\setminus \bar{\Omega }. \end{array}\right.$$
  Then
$$u=G\left(f\right)=G_{\frac{s}{k}}\circ \text{…}\circ G_{\frac{s}{k}}\left(f\right),$$
  where $G_{\frac{s}{k}}$ is the Green operator of ${\left(-\Delta \right)}_{\mathrm{RFL}}^{\frac{s}{k}}$. It seems that G is self‐adjoint and, for 1/2<s<1, we expect its kernel to be of the form
$$G\left(x,y\right)≍{\left|x-y\right|}^{2s-n}{\left(\frac{\delta (x)\delta (y)}{{\left|x-y\right|}^{2}}\wedge 1\right)}^{\frac{s}{k}},\;x,y\in \Omega .$$
(5) The operators that admit exterior data u=g in $R^{n}\setminus \bar{\Omega }$ (for example, the RFL) have an exterior kernel, that is sometimes denoted by P(x,y), $x\in \Omega ,y\in R^{n}\setminus \bar{\Omega }$ (in the case of the RFL it holds that $P\left(x,y\right)=-{\left(-\Delta \right)}_{y}^{s}G\left(x,y\right)$). It seems reasonable that the singular solutions of type $u^{★}$ can also be detected from the outside, as it has been done, for instance, in [6, Lemma 7] and [1, Lemma 3.6].
(6) Note that, so far, we have given all our estimates in terms of $u/u^{★}$. However, it would be nice to give an operator $\hat{M}$ such that
$$\lim_{x\to z}\frac{\hat{M}\left(h\right)\left(x\right)}{\delta {\left(x\right)}^{2s-\gamma -1}}=h\left(z\right),\;z\in \partial \Omega .$$
  Nevertheless, the boundary behaviour of $u^{★}$ is only known in terms of rate. An interesting question is if the following limit is defined
(5.1) $$K\left(z\right)=\lim_{x\to z}\frac{u^{★}\left(x\right)}{\delta {\left(x\right)}^{2s-\gamma -1}},\;z\in \partial \Omega .$$
  This seems to be a further assumption on the kernel. If it is, then K≍1, we can set
$$\hat{M}\left(h\right)=M\left(\frac{h}{K}\right),$$
  so that
$$\lim_{x\to z}\frac{\hat{M}\left(h\right)\left(x\right)}{\delta {\left(x\right)}^{2s-\gamma -1}}=\lim_{x\to z}\frac{M(h/K)(x)}{u^{★}\left(x\right)}\frac{u^{★}\left(x\right)}{\delta {\left(x\right)}^{2s-\gamma -1}}=\frac{h(z)}{K(z)}K\left(z\right)=h\left(z\right).$$
  As it was pointed to us by Grubb, such operator $\hat{M}$ for the RFL has been provided in [30, Corollary 6.2 and Theorem 7.1]. Also, as a matter of fact, always in the RFL case, the function K defined in (5.1) is constant: this is a consequence of the equivalence between the integration by parts formula in [32, Corollary 4.5] and the one in [1, Proposition 2].
(7) In the case of the RFL, the existence of solutions which are singular at boundary can be obtained by taking the derivative of regular solutions. In Appendix A, we include an account of how positive singular solutions can be obtained, which was explained to us by Ros‐Oton. It is based on a very interesting formula of chain‐rule type, see (A.1).
  However, this argument does not seem to apply in general. In particular, it could fail in those examples where the commutation with the derivative does not hold, so that the singular rates cannot be predicted by such means. For the SFL it is easy to see that we cannot repeat the reasoning: in one dimension (say Ω=(−1,1)), we take the first eigenfunction for the SFL $u\left(x\right)=\cos\frac{\pi x}{2}$. Then, all derivatives are bounded functions and no singularity appears. However, we have shown that the blow‐up rate of the critical solution is $\delta^{2(s-1)}$.
(8) It is interesting to point out that, when $f≍\delta^{-2s}$ (and it is admissible in the sense of (1.4), that is, 2s<γ+1), then our main result Theorem 3.4 says that G(f)≍1.
  Given $g\in L^{\infty }\left(\partial \Omega \right)\;$ it is therefore natural to ask whether there exists a function f such that G(f)(x)→g(z) as x→z∈∂Ω.
  This would amount to studying whether the non‐homogeneous Dirichlet problem
$$\left\{\begin{array}{cccc} Lu & =f & & \text{in}\;\Omega \\ u & =g & & \text{on}\;\partial \Omega \\ u & =0 & & \text{in}\;R^{n}\setminus \bar{\Omega }\;\text{(if}\;\text{applicable)}\; \end{array}\right.$$
  has solutions for g bounded. When 2s−γ−1<0 the solution of such problem will satisfy $\lim_{x\to \partial \Omega }u/u^{★}=0$ on ∂Ω. This would indicate that u=G(f). If such u exists, it will never be unique since, taking $f_{2}\in C_{c}^{\infty }\left(\Omega \right)$, $\hat{u}=G\left(f+f_{2}\right)=u+G\left(f_{2}\right)$ will also go to g at the boundary. Hence, it seems that, for operators L with a Green kernel satisfying 2s−γ−1<0, f and g cannot be chosen independently. In fact, for compactly supported f the only possible bounded g is zero, in complete contrast to the problem for the classical Laplacian.
  In many cases, this inverse task of finding one or several f given g turns out to be simple. If, for instance, the direct operator L is given by a singular integral, then given some bounded smooth boundary data g we can extend them to the interior of Ω as a smooth function $\tilde{g}$, and by zero outside. Then, we can take $u=\tilde{g}$ and compute f=Lu in Ω explicitly, for Ω of class $C^{1,1}$. This construction is particularly enlightening when g=1. The natural extension to the inside is
$$u\left(x\right)=\tilde{g}\left(x\right)=\left\{\begin{array}{cc} 1 & \text{in}\;x\in \bar{\Omega },\\ 0 & \text{in}\;x\in R^{n}\setminus \bar{\Omega }. \end{array}\right.$$
  When L is the RFL, the computation yields, for x∈Ω,
$$f_{\mathrm{RFL}}\left(x\right)={\left(-\Delta \right)}_{\mathrm{RFL}}^{s}u\left(x\right)=c_{n,s}\;p.v.\;\int_{\Omega }\frac{1-1}{{\left|x-y\right|}^{n+2s}}\;dy+c_{n,s}\int_{R^{n}\setminus \bar{\Omega }}\frac{1-0}{{\left|x-y\right|}^{n+2s}}\;dy≍\delta {\left(x\right)}^{-2s}.$$
  The last computation is a simple although technical exercise. Note that f is in $L^{1}\left(\Omega \right)$ if s<1/2 and in $L^{2}\left(\Omega \right)$ if  s<1/4.
  For the SFL, we use the kernel representation and deduce, for x∈Ω,
$$\begin{array}{c} f_{\mathrm{SFL}}\left(x\right)={\left(-\Delta \right)}_{\mathrm{SFL}}^{s}u\left(x\right)=p.v.\;\int_{\Omega }\left(1-1\right)\;J\left(x,y\right)\;dy+\kappa \left(x\right)=\kappa \left(x\right)≍\delta {\left(x\right)}^{-2s}, \end{array}$$
  cf. (1.9). Curiously, in the CFL (which satisfies 2s−γ−1=0) case, the L‐harmonic problem has $u^{★}≍1$ and we get the non‐homogeneous Dirichlet problem. Thus, for u=1 one trivially has for all x∈Ω,
$$f_{\mathrm{CFL}}\left(x\right)={\left(-\Delta \right)}_{\mathrm{CFL}}^{s}u\left(x\right)=c_{n,s}\;p.v.\;\int_{\Omega }\frac{\left(1-1\right)}{{\left|x-y\right|}^{n+2s}}dy=0.$$
  This shows, in particular, that u=1 is CFL‐harmonic.
(9) In this paper, we present optimal *function* data (and, under additional assumptions, measures). It is known that the RFL, SFL, and CFL not only improve integrability, but also differentiability, that is, solutions for $L^{p}$ data are in Sobolev spaces $W^{t,q}$ for some t>0 and p⩾q. Then, it is natural to consider distributional data $f\in W^{-t,q}$ in the weak‐dual formulation. Some results in this direction are due to Grubb [29, 30].
  Our techniques do not extend to distributional solutions, since we have used a theory of non‐negative data: in particular, we make use of the positive part $f_{+}$ which is not defined on distributions. This is not surprising, for our assumptions on the kernel are upper and lower bounds (and possibly continuity) but never differentiability.
(10) It would interesting to see under which conditions the results that hold for $h\in L^{1}\left(\partial \Omega \right)$ (notably, Theorems 4.6 and 4.15) can be extended to h∈M(∂Ω).
(11) Finally, we would like to stress that we have dealt here only with linear equations. In a nonlinear setting the situation may be even richer, as boundary singularities could be generated by the nonlinearity as remarked, for example, in [1] for the RFL and in [2] for the SFL: typically this type of behaviour is not captured by a Green‐Martin representation as the one in (K0) and (4.9), because the boundary blow‐up rate of these solutions exceeds the one of the reference function $u^{*}$ as built in Theorem 4.1.

## JOURNAL INFORMATION

The *Journal of the London Mathematical Society* is wholly owned and managed by the London Mathematical Society, a not‐for‐profit Charity registered with the UK Charity Commission. All surplus income from its publishing programme is used to support mathematicians and mathematics research in the form of research grants, conference grants, prizes, initiatives for early career researchers and the promotion of mathematics.

## Footnotes

† The space $L_{c}^{\infty }\left(\Omega \right)$ is the subspace of $L^{\infty }\left(\Omega \right)$ made up of functions with compact support in Ω.

† Recall Remark 3.1 for the definition of ε.

## APPENDIX A DERIVATION OF AN EXPLICIT SINGULAR SOLUTION BY X. ROS‐OTON

This section is the result of conversations with Prof. X. Ros‐Oton who had indicated to the authors that, at least in the RFL case, some singular solutions could be obtained by differentiating the continuous solutions of the standard theory as developed in [38], see also comment number 7 in Section 5. The objection we made that the solutions obtained by plain differentiation may change sign was taken into account. In this section, for the sake of clarity we drop the subindex RFL: ${\left(-\Delta \right)}^{s}={\left(-\Delta \right)}_{\mathrm{RFL}}^{s}$.

This is the way his argument proceeds in four steps. Working in arbitrary dimension, we consider functions $u\in W^{1,1}\left(R^{N}\right)$. First, we need the interesting identity

(A.1)
$${\left(-\Delta \right)}^{s}\left(x\cdot \nabla u\right)=x\cdot \nabla {\left(-\Delta \right)}^{s}u+2s{\left(-\Delta \right)}^{s}u,\;\text{in}\;\;R^{n}.$$

This identity is proved in [38, Proof of Lemma 5.1] by calculations with integrals, but we suggest the reader to do it as an exercise, by taking Fourier transforms and manipulating the resulting formula.

Then we need the result by Getoor (see [27, section 3] or [4]) that applies to a very particular continuous solution

$${\left(-\Delta \right)}^{s}{\left(1-{\left|x\right|}^{2}\right)}_{+}^{s}=C>0\;\text{in}\;B_{1}\subset R^{n}$$

with C=C(n,s)>0 (see also [24, 25]). The next step is new and goes as follows. If we call $U\left(x\right)={\left(1-{\left|x\right|}^{2}\right)}_{+}^{s}$ and put V=x·∇U we conclude that

$${\left(-\Delta \right)}^{s}V={\left(-\Delta \right)}^{s}\left(x\cdot \nabla U\right)=x\cdot \nabla C+2sC=2sC\;\text{in}\;B_{1}.$$

Finally, we consider W=2sU−V and get ${\left(-\Delta \right)}^{s}W=0$ in $B_{1}$. Moreover,

$$W=2sU-x\cdot \nabla U=\frac{2s(1-|x{|}^{2})}{{\left(1-{\left|x\right|}^{2}\right)}^{1-s}}+\frac{{2s|x|}^{2}}{{\left(1-{\left|x\right|}^{2}\right)}^{1-s}}=\frac{2s}{{\left(1-{\left|x\right|}^{2}\right)}^{1-s}}\;\text{in}\;B_{1},$$

and this is a singular solution up to a harmless constant. This is precisely the singular solution provided by Hmissi [33] and Bogdan [6].

## APPENDIX B PROOFS OF LEMMAS 3.2 AND 3.3

Proof of Lemma 3.2 We estimate

$$\begin{array}{ccc} G\left(\delta^{\beta }\chi_{\left\{\delta <\eta \right\}}\right)\left(x\right) & ≍ & \int_{\left\{\delta (y)<\eta \right\}}\frac{\delta {\left(y\right)}^{\beta }}{{\left|x-y\right|}^{n-2s}}{\left(\frac{\delta (x)\delta (y)}{{\left|x-y\right|}^{2}}\wedge 1\right)}^{\gamma }\;dy\\ & ≍ & \delta {\left(x\right)}^{\gamma }\int_{\left\{\delta (y)<\eta /4\right\}}\frac{\delta {\left(y\right)}^{\beta +\gamma }}{{\left|x-y\right|}^{n-2s+2\gamma }}\;dy+\int_{\left\{\eta /4<\delta (y)<\eta \right\}}\frac{\delta {\left(y\right)}^{\beta }}{{\left|x-y\right|}^{n-2s}}\;dy. \end{array}$$

Using the co‐area formula (see, for example, [26]), we have that

(B.1)
$$\begin{array}{cc} \int_{\left\{\delta (y)<\eta /4\right\}}\frac{\delta {\left(y\right)}^{\beta +\gamma }}{{\left|x-y\right|}^{n-2s+2\gamma }}\;dy & =\int_{0}^{\eta /4}t^{\beta +\gamma }\int_{\left\{\delta (y)=t\right\}}\frac{dy}{{\left|x-y\right|}^{n-2s+2\gamma }}\;dt, \end{array}$$

(B.2)
$$\begin{array}{cc} \int_{\left\{\eta /4<\delta (y)<\eta \right\}}\frac{\delta {\left(y\right)}^{\beta }}{{\left|x-y\right|}^{n-2s}}\;dy & =\int_{\eta /4}^{\eta }t^{\beta }\int_{\left\{\delta (y)=t\right\}}\frac{dy}{{\left|x-y\right|}^{n-2s}}\;dt. \end{array}$$

Let us first deal with (B.1). The inner (n−1)‐dimensional integral is uniformly bounded in η whenever −2s+2γ<−1, which is $\gamma <s-\frac{1}{2}$. The integration in the t variable concludes then the claimed estimate. If instead $\gamma >s-\frac{1}{2}$, then we have

$$\begin{array}{cc} \int_{\left\{\delta (y)<\eta /4\right\}}\frac{\delta {\left(y\right)}^{\beta +\gamma }}{{\left|x-y\right|}^{n-2s+2\gamma }}\;dy & ≍\;\int_{0}^{\eta /4}t^{\beta +\gamma }{\left(\delta (x)-t\right)}^{2s-2\gamma -1}\;dt\\ & ≍\;\delta {\left(x\right)}^{\beta -\gamma +2s}\int_{0}^{\eta /\delta (x)}t^{\beta +\gamma }{\left(4-t\right)}^{2s-2\gamma -1}dt\\ & ≍\;\delta {\left(x\right)}^{\beta -\gamma +2s}{\left(\frac{\eta }{\delta (x)}\right)}^{\beta +\gamma +1}=\eta^{\beta +\gamma +1}\delta {\left(x\right)}^{2s-2\gamma -1}; \end{array}$$

as in this case it is 2s−2γ−1<0, then we conclude

$$\begin{array}{c} \int_{\left\{\delta (y)<\eta /4\right\}}\frac{\delta {\left(y\right)}^{\beta +\gamma }}{{\left|x-y\right|}^{n-2s+2\gamma }}\;dy⩽\eta^{\beta +2s-\gamma } \end{array}$$

up to universal constants. Needless to say, the above also holds when $\gamma =s-\frac{1}{2}$ with the suitable modifications and we get

$$\begin{array}{c} \int_{\left\{\delta (y)<\eta /4\right\}}\frac{\delta {\left(y\right)}^{\beta +\gamma }}{{\left|x-y\right|}^{n-2s+2\gamma }}\;dy⩽\eta^{\beta +\gamma +1}\;\left|\;\ln\eta \right|. \end{array}$$

Let us now give a close look at (B.2). The inner (n−1)‐dimensional integral is uniformly bounded in η whenever $s>\frac{1}{2}$, then the integration in t is elementary. If s<1/2, reasoning as above we get

$$\begin{array}{c} \int_{\left\{\eta /4<\delta (y)<\eta \right\}}\frac{\delta {\left(y\right)}^{\beta }}{{\left|x-y\right|}^{n-2s}}\;dy≍\int_{\eta /4}^{\eta }t^{\beta }|\delta \left(x\right)-t{|}^{2s-1}\;dt≍\eta^{\beta }\delta {\left(x\right)}^{2s}, \end{array}$$

and, in case s=1/2,

$$\begin{array}{c} \int_{\left\{\eta /4<\delta (y)<\eta \right\}}\frac{\delta {\left(y\right)}^{\beta }}{{\left|x-y\right|}^{n-2s}}\;dy≍\eta^{\beta }\delta \left(x\right)\;\left|\;\ln\delta \left(x\right)\right|. \end{array}$$

The above proves the first claim in the statement. Mind now that in the case $\gamma <s-\frac{1}{2}$ we could simply estimate

$$\begin{array}{c} G\left(\delta^{\beta }\chi_{\left\{\delta <\eta \right\}}\right)\left(x\right)⩽\delta {\left(x\right)}^{\gamma }\int_{\left\{\delta (y)<\eta \right\}}\frac{\delta {\left(y\right)}^{\beta +\gamma }}{{\left|x-y\right|}^{n-2s+2\gamma }}\;dy \end{array}$$

and the above analysis would then bear

$$G\left(\delta^{\beta }\chi_{\left\{\delta <\eta \right\}}\right)\left(x\right)⩽\eta^{\beta +\gamma +1}\delta {\left(x\right)}^{\gamma },\;\text{for}\;\delta \left(x\right)>\eta /2.$$

□

Proof of Lemma 3.3 From now on let x∈{δ<η/2} be fixed. Call Φ:B(x,1)→B(0,1) a diffeomorphism satisfying

(B.3)
$$\begin{array}{c} \begin{array}{cc} & \Phi \left(\Omega \cap B\left(x,1\right)\right)=B\left(0,1\right)\cap \left\{y\in R^{n}:y\cdot e_{n}>0\right\}\\ & \Phi \left(y\right)\cdot e_{n}=\delta \left(y\right)\;\text{for}\;\text{any}\;y\in B\left(x,1\right),\;\Phi \left(x\right)=\delta \left(x\right)e_{n}. \end{array} \end{array}$$

Also, we are going to intensively use (K0), (K1), and (K2). We split the estimate into the five regions

$$\begin{array}{cc} \Omega_{1}:=B\left(x,\delta (x)/2\right),\; & \Omega_{2}:=\left\{y:\delta \left(y\right)<\eta \right\}\setminus B\left(x,1\right),\\ \Omega_{3}:=\left\{y:\delta \left(y\right)<\delta \left(x\right)/2\right\}\cap B\left(x,1\right),\; & \Omega_{4}:=\left\{y:3\delta \left(x\right)/2<\delta \left(y\right)<\eta \right\}\cap B\left(x,1\right),\\ \Omega_{5}:=\left\{y:\delta \left(x\right)/2<\delta \left(y\right)<3\delta \left(x\right)/2\right\} & \cap \left(B(x,1)\setminus B(x,\delta (x)/2)\right). \end{array}$$

- For $y\in \Omega_{1}$ we use that
  $$\begin{array}{c} \left(\frac{\delta (x)\delta (y)}{{\left|x-y\right|}^{2}}\wedge 1\right)≍1, \end{array}$$
  so that
  $$\begin{array}{cc} \int_{\Omega_{1}}G\left(x,y\right)\;\delta {\left(y\right)}^{\beta }\;dy & ≍\int_{\Omega_{1}}\frac{\delta {\left(y\right)}^{\beta }}{{\left|x-y\right|}^{n-2s}}\;dy\\ & ≍\delta {\left(x\right)}^{\beta }\int_{B(x,\delta (x)/2)}\frac{dy}{{\left|x-y\right|}^{n-2s}}≍\delta {\left(x\right)}^{\beta +2s}. \end{array}$$
- For $y\in \Omega_{2}$ we use that
  $$\begin{array}{c} \left(\frac{\delta (x)\delta (y)}{{\left|x-y\right|}^{2}}\wedge 1\right)≍\frac{\delta (x)\delta (y)}{{\left|x-y\right|}^{2}}, \end{array}$$
  so that
  $$\begin{array}{cc} \int_{\Omega_{2}}G\left(x,y\right)\delta {\left(y\right)}^{\beta }dy & ≍\delta {\left(x\right)}^{\gamma }\int_{\Omega_{2}}\frac{\delta {\left(y\right)}^{\beta +\gamma }}{{\left|x-y\right|}^{n-2s+2\gamma }}dy\\ & ≍\;\delta {\left(x\right)}^{\gamma }\int_{\Omega_{2}}\delta {\left(y\right)}^{\beta +\gamma }dy\;≍\;\eta^{\beta +\gamma +1}\delta {\left(x\right)}^{\gamma }. \end{array}$$
- For $y\in \Omega_{3}$ we use that
  $$\begin{array}{c} \left(\frac{\delta (x)\delta (y)}{{\left|x-y\right|}^{2}}\wedge 1\right)≍\frac{\delta (x)\delta (y)}{{\left|x-y\right|}^{2}}, \end{array}$$
  so that
  $$\begin{array}{c} \int_{\Omega_{3}}G\left(x,y\right)\;\delta {\left(y\right)}^{\beta }\;dy≍\delta {\left(x\right)}^{\gamma }\int_{\Omega_{3}}\frac{\delta {\left(y\right)}^{\beta +\gamma }}{{\left|x-y\right|}^{n-2s+2\gamma }}\;dy. \end{array}$$
  Applying the change of variable entailed by Φ — as defined in (B.3) — we get
  $$\begin{array}{cc} & \int_{\Omega_{3}}G\left(x,y\right)\delta {\left(y\right)}^{\beta }dy≍\delta {\left(x\right)}^{\gamma }\int_{\{0<z_{n}<\delta \left(x\right)/2\}\cap B\left(0,1\right)}\frac{z_{n}^{\beta +\gamma }}{{\left(|\delta \left(x\right)-z_{n}\left|+\right|z^{\prime }|\right)}^{n-2s+2\gamma }}\;dz\\ & \;≍\;\delta {\left(x\right)}^{\gamma }\int_{\left\{|z^{\prime }|<1\right\}}\int_{0}^{\delta (x)/2}\frac{z_{n}^{\beta +\gamma }}{{\left(|\delta \left(x\right)-z_{n}\left|+\right|z^{\prime }|\right)}^{n-2s+2\gamma }}\;dz_{n}\;dz^{\prime }\\ & \;≍\;\delta {\left(x\right)}^{\gamma }\int_{0}^{1}t^{n-2}\int_{0}^{\delta (x)/2}\frac{z_{n}^{\beta +\gamma }}{{\left((\delta \left(x\right)-z_{n})+t\right)}^{n-2s+2\gamma }}\;dz_{n}\;dt\\ & \;≍\;\delta {\left(x\right)}^{\beta +2s}\int_{0}^{1/\delta (x)}t^{n-2}\int_{0}^{1/2}\frac{h^{\beta +\gamma }}{{\left((1-h)+t\right)}^{n-2s+2\gamma }}\;dh\;dt\\ & \;≍\;\delta {\left(x\right)}^{\beta +2s}\int_{0}^{1/\delta (x)}\frac{t^{n-2}}{{\left(1+t\right)}^{n-2s+2\gamma }}\;dt≍\;\delta {\left(x\right)}^{\beta +2s}\int_{0}^{1/\delta (x)}\frac{dt}{{\left(1+t\right)}^{2-2s+2\gamma }}\\ & \;≍\;\delta {\left(x\right)}^{\beta +2s}\;\left\{\begin{array}{cccc} & 1 & & \text{if}\;1-2s+2\gamma >0\\ & \left|\;\ln(\delta (x))\right| & & \text{if}\;1-2s+2\gamma =0\\ & \delta {\left(x\right)}^{1-2s+2\gamma } & & \text{if}\;1-2s+2\gamma <0, \end{array}\right. \end{array}$$
  which, rephrased, means
  $$\begin{array}{c} \int_{\Omega_{3}}G\left(x,y\right)\;\delta {\left(y\right)}^{\beta }\;dy≍\left\{\begin{array}{cccc} & \delta {\left(x\right)}^{\beta +2s} & & \text{if}\;\gamma >s-\frac{1}{2},\\ & \delta {\left(x\right)}^{\beta +2s}\;\left|\;\ln\left(\delta \left(x\right)\right)\right| & & \text{if}\;\gamma =s-\frac{1}{2},\\ & \delta {\left(x\right)}^{\beta +2\gamma +1} & & \text{if}\;\gamma <s-\frac{1}{2}. \end{array}\right. \end{array}$$
- For the integration in $\Omega_{4}$, we use that, for $y\in \Omega_{4}$,
  $$\begin{array}{c} \left(\frac{\delta (x)\delta (y)}{{\left|x-y\right|}^{2}}\wedge 1\right)≍\frac{\delta (x)\delta (y)}{{\left|x-y\right|}^{2}}, \end{array}$$
  so that
  $$\begin{array}{cc} & \int_{\Omega_{4}}G\left(x,y\right)\;\delta {\left(y\right)}^{\beta }\;dy≍\delta {\left(x\right)}^{\gamma }\int_{\left\{3\delta (x)/2<\delta (y)<\eta \}\cap B(x,1\right)}\frac{\delta {\left(y\right)}^{\beta +\gamma }}{|x-y{|}^{n-2s+2\gamma }}\;dy \end{array}$$
  and, changing variables at the aid of Φ as above,
  $$\begin{array}{cc} \int_{\Omega_{3}}G\left(x,y\right)\;\delta {\left(y\right)}^{\beta }\;dy & ≍\delta {\left(x\right)}^{\gamma }\int_{\{3\delta \left(x\right)/2<z_{n}<\eta \}\cap B\left(0,1\right)}\frac{z_{n}^{\beta +\gamma }}{{\left(|\delta \left(x\right)-z_{n}\left|+\right|z^{\prime }|\right)}^{n-2s+2\gamma }}\;dz\\ & ≍\;\delta {\left(x\right)}^{\beta +2s}\int_{3/2}^{\eta /\delta (x)}\int_{0}^{1/\delta (x)}\frac{h^{\beta +\gamma }\;t^{n-2}}{{\left((h-1)+t\right)}^{n-2s+2\gamma }}\;dt\;dh\\ & ≍\;\delta {\left(x\right)}^{\beta +2s}\int_{3/2}^{\eta /\delta (x)}\int_{0}^{1/((h-1)\delta (x))}\frac{h^{\beta +\gamma }\;r^{n-2}}{{\left(h-1\right)}^{1-2s+2\gamma }{\left(1+r\right)}^{n-2s+2\gamma }}\;dr\;dh\\ & ≍\;\delta {\left(x\right)}^{\beta +2s}\int_{3/2}^{\eta /\delta (x)}\frac{h^{\beta +\gamma }}{{\left(h-1\right)}^{1-2s+2\gamma }}\int_{1}^{1/((h-1)\delta (x))}\frac{dr}{{\left(1+r\right)}^{2-2s+2\gamma }}\;dh. \end{array}$$
  At this point we need to separate into different cases, since
  $$\int_{1}^{1/((h-1)\delta (x))}\frac{dr}{{\left(1+r\right)}^{2-2s+2\gamma }}≍\left\{\begin{array}{cccc} & 1 & & \text{if}\;1-2s+2\gamma >0\\ & \left|\;\ln((h-1)\delta (x))\right| & & \text{if}\;1-2s+2\gamma =0\\ & {\left(h-1\right)}^{1-2s+2\gamma }\delta {\left(x\right)}^{1-2s+2\gamma } & & \text{if}\;1-2s+2\gamma <0, \end{array}\right.$$
  which, rephrased, gives
  $$\begin{array}{cc} \int_{\Omega_{3}}G\left(x,y\right)\;\delta {\left(y\right)}^{\beta }\;dy & ≍\;\delta {\left(x\right)}^{\beta +2s}\;\left\{\begin{array}{cccc} & \int_{3/2}^{\eta /\delta (x)}\frac{h^{\beta +\gamma }}{{\left(h-1\right)}^{1-2s+2\gamma }}\;dh & & \text{if}\;\gamma >s-\frac{1}{2}\\ & \int_{3/2}^{\eta /\delta (x)}h^{\beta +\gamma }\left|\;\ln\left(\left(h-1\right)\delta \left(x\right)\right)\right|\;dh\;\; & & \text{if}\;\gamma =s-\frac{1}{2}\\ & \delta {\left(x\right)}^{1-2s+2\gamma }\int_{3/2}^{\eta /\delta (x)}h^{\beta +\gamma }\;dh & & \text{if}\;\gamma <s-\frac{1}{2} \end{array}\right.\\ & \;≍\;\left\{\begin{array}{cccc} & \delta {\left(x\right)}^{\beta +2s}\int_{3/2}^{\eta /\delta (x)}h^{\beta -\gamma +2s-1}dh & & \text{if}\;\gamma >s-\frac{1}{2}\\ & \delta {\left(x\right)}^{\beta +2s}\int_{3/2}^{\eta /\delta (x)}h^{\beta +\gamma }\left|\;\ln\left(\left(h-1\right)\delta \left(x\right)\right)\right|\;dh\; & & \text{if}\;\gamma =s-\frac{1}{2}\\ & \delta {\left(x\right)}^{\beta +2\gamma +1}\int_{3/2}^{\eta /\delta (x)}h^{\beta +\gamma }\;dh & & \text{if}\;\gamma <s-\frac{1}{2}. \end{array}\right. \end{array}$$
  Now, in the case $\gamma >s-\frac{1}{2}$ we have
  $$\begin{array}{cc} \int_{\Omega_{3}}G\left(x,y\right)\delta {\left(y\right)}^{\beta }dy & ≍\delta {\left(x\right)}^{\beta +2s}\left\{\begin{array}{cccc} & \eta^{\beta -2\gamma +2s}\delta {\left(x\right)}^{-\beta +\gamma -2s} & & \text{if}\;\beta -\gamma +2s>0\\ & \left|\;\ln(\eta /\delta (x))\right| & & \text{if}\;\beta -\gamma +2s=0\\ & 1 & & \text{if}\;\beta -\gamma +2s<0 \end{array}\right.\\ & =\left\{\begin{array}{cccc} & \eta^{\beta -2\gamma +2s}\delta {\left(x\right)}^{\gamma } & & \text{if}\;\beta -\gamma +2s>0\\ & \delta {\left(x\right)}^{\beta +2s}\left|\;\ln\left(\eta /\delta \left(x\right)\right)\right| & & \text{if}\;\beta -\gamma +2s=0\\ & \delta {\left(x\right)}^{\beta +2s} & & \text{if}\;\beta -\gamma +2s<0, \end{array}\right. \end{array}$$
  when $\gamma =s-\frac{1}{2}$
  $$\begin{array}{cc} & \int_{\Omega_{3}}G\left(x,y\right)\;\delta {\left(y\right)}^{\beta }\;dy≍\delta {\left(x\right)}^{\beta +2s}\int_{3/2}^{\eta /\delta (x)}h^{\beta +\gamma }\left|\;\ln\left(\left(h-1\right)\delta \left(x\right)\right)\right|\;dh\\ & \;≍\;\delta {\left(x\right)}^{2s-\gamma -1}\eta^{\beta +\gamma +1}|\;\ln\left(\left(\eta /\delta \left(x\right)-1\right)\delta \left(x\right)\right)|\;≍\;\eta^{\beta +\gamma +1}\;\left|\;\ln\eta \right|\;\delta {\left(x\right)}^{\gamma }, \end{array}$$
  whereas for $\gamma <s-\frac{1}{2}$ we have
  $$\begin{array}{c} \int_{\Omega_{3}}G\left(x,y\right)\;\delta {\left(y\right)}^{\beta }\;dy≍\delta {\left(x\right)}^{\beta +2\gamma +1}{\left(\frac{\eta }{\delta (x)}\right)}^{\beta +\gamma +1}=\eta^{\beta +\gamma +1}\delta {\left(x\right)}^{\gamma }. \end{array}$$
  Resuming the information collected about the integral over $\Omega_{4}$, we have the behaviour described in Table B.1.
- For $y\in \Omega_{5}$ we use that
  $$\begin{array}{c} \left(\frac{\delta (x)\delta (y)}{{\left|x-y\right|}^{2}}\wedge 1\right)≍\frac{\delta (x)\delta (y)}{{\left|x-y\right|}^{2}}, \end{array}$$
  so that
  $$\begin{array}{cc} \int_{\Omega_{5}}G\left(x,y\right)\;\delta {\left(y\right)}^{\beta }\;dy & ≍\delta {\left(x\right)}^{\gamma }\int_{\left\{\delta (x)/2<\delta (y)<3\delta (x)/2\}\cap (B(x,1)\setminus B(x,\delta (x)/2)\right)}\frac{\delta {\left(y\right)}^{\beta +\gamma }}{|x-y{|}^{n-2s+2\gamma }}\;dy\\ & ≍\;\delta {\left(x\right)}^{\beta +2\gamma }\int_{\left\{\delta (x)/2<\delta (y)<3\delta (x)/2\}\cap (B(x,1)\setminus B(x,\delta (x)/2)\right)}|x-y{|}^{-n+2s-2\gamma }\;dy \end{array}$$
  and, applying the change of variable induced by the Φ defined in (B.3),
  $$\begin{array}{cc} \int_{\Omega_{5}}G\left(x,y\right)\;\delta {\left(y\right)}^{\beta }\;dy & ≍\delta {\left(x\right)}^{\beta +2\gamma }\int_{\delta (x)/2}^{3\delta (x)/2}\int_{\delta (x)/2}^{1}r^{n-2}{\left(|\delta (x)-h|+r\right)}^{-n+2s-2\gamma }\;dr\;dh\\ & ≍\;\delta {\left(x\right)}^{\beta +2\gamma }\int_{\delta (x)/2}^{1}r^{2s-2\gamma -1}\int_{-\delta (x)/(2r)}^{\delta (x)/(2r)}{\left(|t|+1\right)}^{-n+2s-2\gamma }\;dt\;dr\\ & ≍\;\delta {\left(x\right)}^{\beta +2s}\int_{1/2}^{1/\delta (x)}\rho^{2s-2\gamma -1}\int_{0}^{1/\rho }{\left(t+1\right)}^{-n+2s-2\gamma }\;dt\;d\rho \\ & ≍\;\delta {\left(x\right)}^{\beta +2s}\int_{1/2}^{1/\delta (x)}\rho^{2s-2\gamma -2}\;d\rho \\ & ≍\delta {\left(x\right)}^{\beta +2s}\left\{\begin{array}{cccc} & \delta {\left(x\right)}^{-2s+2\gamma +1} & & \text{if}\;2s-2\gamma -1>0\\ & \left|\;\ln\delta (x)\right| & & \text{if}\;2s-2\gamma -1=0\\ & 1 & & \text{if}\;2s-2\gamma -1<0 \end{array}\right. \end{array}$$
  meaning
  $$\begin{array}{c} \int_{\Omega_{5}}G\left(x,y\right)\;\delta {\left(y\right)}^{\beta }\;dy\;≍\;\left\{\begin{array}{cccc} & \delta {\left(x\right)}^{\beta +2\gamma +1} & & \text{if}\;\gamma <s-\frac{1}{2}\\ & \delta {\left(x\right)}^{\beta +2s}\left|\;\ln\delta \left(x\right)\right| & & \text{if}\;\gamma =s-\frac{1}{2}\\ & \delta {\left(x\right)}^{\beta +2s} & & \text{if}\;\gamma >s-\frac{1}{2}. \end{array}\right. \end{array}$$
  □

**TABLE B.1:** The integration over $\Omega_{4}$

	$\gamma <s-\frac{1}{2}$	$\gamma =s-\frac{1}{2}$	$\gamma >s-\frac{1}{2}$
β<γ−2s	$\eta^{\beta +\gamma +1}\delta {\left(x\right)}^{\gamma }$	$\eta^{\beta +\gamma +1}\;\left|\;\ln\eta \right|\;\delta {\left(x\right)}^{\gamma }$	$\delta {\left(x\right)}^{\beta +2s}$
β=γ−2s	$\eta^{\beta +\gamma +1}\delta {\left(x\right)}^{\gamma }$	$\eta^{\beta +\gamma +1}\;\left|\;\ln\eta \right|\;\delta {\left(x\right)}^{\gamma }$	$\delta {\left(x\right)}^{\beta +2s}\left|\;\ln\left(\eta /\delta \left(x\right)\right)\right|$
β>γ−2s	$\eta^{\beta +\gamma +1}\delta {\left(x\right)}^{\gamma }$	$\eta^{\beta +\gamma +1}\;\left|\;\ln\eta \right|\;\delta {\left(x\right)}^{\gamma }$	$\eta^{\beta -2\gamma +2s}\delta {\left(x\right)}^{\gamma }$
